# A Linear Dysprosium(II) Metallocene with a High Effective Energy Barrier and Magnetic Hysteresis up to 70 Kelvin

**DOI:** 10.1021/jacs.5c06222

**Published:** 2025-05-15

**Authors:** Ming Liu, Yan-Cong Chen, Huan Wang, Tao Shang, Ming-Liang Tong, Richard A. Layfield, Akseli Mansikkamäki, Fu-Sheng Guo

**Affiliations:** † Institute of Fundamental and Frontier Sciences, 12599 University of Electronic Science and Technology of China, Xiyuan Avenue 2006, Chengdu 611731, China; ‡ Key Laboratory of Bioinorganic and Synthetic Chemistry of the Ministry of Education, School of Chemistry, IGCME, GBRCE for Functional Molecular Engineering, Sun Yat-Sen University, Guangzhou 510006, China; § Department of Chemistry, School of Life Sciences, University of Sussex, Brighton BN1 9QR, U.K.; ∥ NMR Research Unit, University of Oulu, P.O. Box 8000, Oulu FI-90014, Finland

## Abstract

Dysprosium in the oxidation state +3 is ubiquitous in studies of single-molecule magnets (SMMs). In contrast, SMMs based on lanthanides in the oxidation state +2 are rare, and examples with both a high effective energy barrier to reversal of the magnetization and hysteresis with coercivity at high temperatures are extremely uncommon. Here, we show that one-electron reduction of the dysprosium­(III) complex [(η^5^-C_5_^*i*^Pr_5_)­Dy­(η^5^-Cp*)­(BH_4_)] (Cp* = C_5_Me_5_) with KC_8_ generates the linear dysprosium­(II) metallocene [(η^5^-C_5_^*i*^Pr_5_)­Dy­(η^5^-Cp*)]. Magnetic measurements, ultraviolet/visible/near infrared (UV/vis/NIR) spectroscopy and theoretical calculations show that the dysprosium­(II) center in [(η^5^-C_5_^*i*^Pr_5_)­Dy­(η^5^-Cp*)] adopts a 4f^9^ (6s/5d)^1^ configuration. Coupling of the 4f and 5d electrons in [(η^5^-C_5_^*i*^Pr_5_)­Dy­(η^5^-Cp*)] results in an effective magnetic moment of 11.38 μ_B_ at 217 K, equaling the highest magnetic moment recorded for a mononuclear complex. AC and DC magnetic measurements establish the SMM properties of [(η^5^-C_5_^*i*^Pr_5_)­Dy­(η^5^-Cp*)], including an energy barrier of 1551 cm^–1^, the largest yet reported for a divalent lanthanide SMM, and a 100-s blocking temperature of 62 K. Magnetic hysteresis measurements produce loops that remain open up to 70 K. Multireference calculations reveal that the eas*y*-axis of magnetization in the ground doublet of [(η^5^-C_5_^*i*^Pr_5_)­Dy­(η^5^-Cp*)] coincides with the molecular symmetry axis, and that this doublet has extremely strong axial character. The strong axiality results in Orbach relaxation probably occurring via the second-excited doublet. The SMM parameters and the theoretical insight suggest that the high effective barrier and blocking temperature are linked to the strong and perfectly axial crystal field experienced by the dysprosium­(II) center.

## Graphical abstract

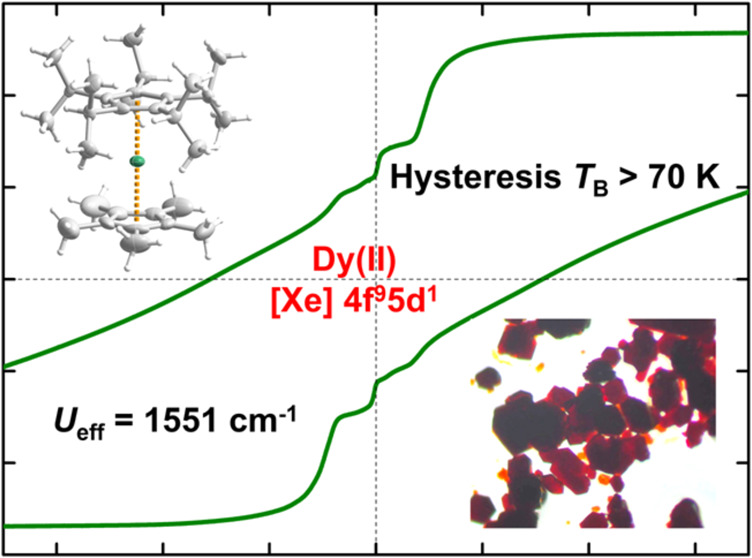

## Introduction

The observation of slow magnetic relaxation in the terbium­(III) complex [TbPc_2_]^−^ (Pc is the phthalocyanine dianion) was a landmark discovery that ushered in a new era in the study of single-molecule magnets (SMMs).^1,2^ An explosion in the number of lanthanide (Ln) SMMs soon followed, with a strong emphasis on trivalent terbium, dysprosium and erbium in addition to other lanthanides.^3–10^ The strong magnetic anisotropy of these Ln^3+^ ions is the key property for observing SMM behavior, when combined with a crystal field of appropriate strength and symmetry.^11–14^ Dysprosium­(III), with its 4f^9^ configuration and large magnetic moment, is by far the most popular lanthanide in single-molecule magnetism. The Kramers nature of Dy^3+^ is important since a bistable magnetic ground is probable almost regardless of the coordination geometry, in contrast to non-Kramers ions such as Tb^3+^, complexes of which must typically adopt strict axial symmetry to act as SMMs.

Early developments with lanthanide SMMs—and dysprosium in particular—were predominantly empirical in nature. More recently, experimental studies of lanthanide SMMs have been informed by multireference calculations, which are often essential for explaining magnetic relaxation phenomena and the role of spin-phonon coupling.^15–21^ The guidelines on how to construct a high-performance SMM based on the oblate electron density of Dy^3+^ are now generally understood to require a strong axial crystal field and a negligible equatorial crystal field, a combination that can produce high effective energy barriers (*U*_eff_) and magnetic hysteresis with coercivity.

One of the most successful manifestations of the “axial” model for dysprosium­(III) SMMs is the cyclopentadienyl approach.^3,22–34^ In particular, metallocene compounds with the general formula [(η^5^-Cp^R^)_2_Dy]­[X], where Cp^R^ is a bulky cyclopentadienyl ligand, such as [C_5_^*i*^Pr_5_]^−^ or [C_5_^*t*^Bu_3_H_2_]^−^, and X denotes a weakly coordinating anion, show some of the highest *U*_eff_ values and hysteresis temperatures.^19,20,35–37^ Indeed, this approach enabled the isolation of the dysprosocenium compound [(η^5^-Cp*)­Dy­(η^5^-C_5_^*i*^Pr_5_)]­[B­(C_6_F_5_)_4_] (Cp* = C_5_Me_5_), the first SMM to show hysteresis above the boiling point of liquid nitrogen.^19^

Compared to the extensive development of SMMs based on trivalent lanthanides, divalent lanthanide SMMs are much less common and are not well understood in terms of magneto-structural correlations. The paucity of divalent lanthanide SMMs is, presumably, related to the challenges associated with the synthesis and isolation of divalent lanthanide compounds in general.^38^ Although the divalent oxidation state is known for all lanthanides (except promethium), the compounds are unstable with respect to oxidation and can be sensitive to solvent and temperature.^39–44^ Despite the drawbacks, the magnetic properties of some nonclassical divalent lanthanide compounds have been described. For example, [K­(2.2.2-cryptand)]­[(η^5^-Cp′)_3_M] (M = Dy, Ho; Cp′ = C_5_H_4_SiMe_3_) show very large effective magnetic moments, thought to originate from the 4f^*n*^5d^1^ configurations (*n* = 9, 10, respectively).^45^ However, slow magnetic relaxation was not reported for these compounds. Beyond SMM properties, divalent lanthanide compounds are also attracting interest for their possible applications in quantum information science.^46,47^

The perfectly linear divalent metallocenes [(η^5^-C_5_^*i*^Pr_5_)_2_M] were reported with M = Tb and Dy, with the terbium­(II) version display a large energy barrier of 1205 cm^–1^ in zero magnetic field along with magnetic hysteresis loops that remain open to 55 K.^48^ In marked contrast, the dysprosium­(II) version did not show any Orbach-type relaxation, hence a *U*_eff_ value could not be determined despite the observation of waist-restricted hysteresis loops up to 75 K for the polycrystalline material. The differing behavior of the two linear metallocenes was attributed to the Kramers nature of terbium­(II) (4f^8^5d^1^) and the non-Kramers nature of dysprosium­(II) (4f^9^5d^1^).

A handful of other divalent lanthanide SMMs has been described, including the thulium­(II) sandwich complex [Tm­(η^8^-COT)_2_{K­(18-crown-6)}_2_], which shows slow magnetic relaxation in zero applied field.^49^ The amidinate-ligated divalent complexes [M­(Piso)_2_] (M = Tb, Dy; Piso = ^*t*^BuC­(NDipp)_2_; Dipp = 2,6-^*i*^Pr_2_C_6_H_3_) show large and remarkably similar *U*_eff_ values of 1334 cm^–1^ and 1365 cm^–1^, respectively, with waist-restricted hysteresis loops at low temperatures.^50^ The near-linear two-coordinate complex [Eu­(N­{SiMePh_2_}_2_)_2_] is an unusual SMM since it is based on the isotropic 4f^7^ ion europium­(II), the energy barrier of 5.6 cm^–1^ apparently arising from magnetic anisotropy related to second-order spin–orbit coupling.^51^ Although not formally divalent, the mixed-valence dinuclear compounds [(C_5_^*i*^Pr_5_)_2_M_2_I_3_] (M = Tb, Dy, Ho) feature an additional d-electron shared by the metal ions, leading to very high energy barriers and unprecedentedly large coercive fields.^52^ Beyond conventional molecular chemistry, the recently reported endohedral metallofullerene Dy_2_@C_82_ consists of two dysprosium­(II) ions confined inside the carbon cage, with strong antiferromagnetic coupling leading to open hysteresis loops up to 25 K.^53^

Intrigued by the lack of an observable barrier in the linear non-Kramers metallocene [(η^5^-C_5_^*i*^Pr_5_)_2_Dy],^48^ we sought to determine if other linear dysprosium­(II) metallocenes would behave in the same way, and to develop a magneto-structural correlation with aid of theoretical calculations to explain the properties. We therefore undertook to synthesize the dysprosocene [(η^5^-C_5_^*i*^Pr_5_)­Dy­(η^5^-Cp*)], that is, the divalent analogue of the dysprosocenium cation [(η^5^-C_5_^*i*^Pr_5_)­Dy­(η^5^-Cp*)]^+^ reported previously by us.^19^ Here, we show that the SMM properties of divalent [(η^5^-C_5_^*i*^Pr_5_)­Dy­(η^5^-Cp*)] are strikingly different to those of [(η^5^-C_5_^*i*^Pr_5_)_2_Dy], and include a large *U*_eff_ value of 1551 cm^–1^ (2232 K) with open magnetic hysteresis loops up to 70 K.

## Results and Discussion

The target compound [(η^5^-C_5_^*i*^Pr_5_)­Dy­(η^5^-Cp*)] was synthesized by reducing the trivalent precursor [(η^5^-C_5_^*i*^Pr_5_)­Dy­(η^5^-Cp*)­(BH_4_)] with 1.5 equiv of KC_8_ in benzene at room temperature over 3 days (Scheme 1). During the reduction, the color of the solution changed from pale yellow to dark red-brown in a few minutes. The red-brown powder obtained after filtration and evaporation of the solvent was recrystallized from hexane at −35 °C to give dark red crystals of [(η^5^-C_5_^*i*^Pr_5_)­Dy­(η^5^-Cp*)]·0.5hexane in 40% yield. Comparing the fourier transform infrared spectroscopy (FTIR) spectra of [(η^5^-C_5_^*i*^Pr_5_)­Dy­(η^5^-Cp*)] and [(η^5^-C_5_^*i*^Pr_5_)­Dy­(η^5^-Cp*)­(BH_4_)] showed that the borohydride ligand is completely removed upon KC_8_ reduction (Figures S1–S2). The synthesis of [(η^5^-C_5_^*i*^Pr_5_)­Dy­(η^5^-Cp*)] can also be achieved in hexane, although longer reaction times are required and the yields are lower. Care must be taken not to add excess KC_8_ to [(η^5^-C_5_^*i*^Pr_5_)­Dy­(η^5^-Cp*)­(BH_4_)] since doing so results in the formation of the triple-decker benzene tetra-anion complex [(η^5^-C_5_^*i*^Pr_5_)­Dy­(μ:η^6^:η^6^-C_6_H_6_)­Dy­(η^5^-C_5_^*i*^Pr_5_)].^54,55^ The dysprosocene [(η^5^-C_5_^*i*^Pr_5_)­Dy­(η^5^-Cp*)] is extremely sensitive to air and moisture but does not show signs of decomposition in the solid-state following storage under argon at room temperature for several months.

**Scheme 1:**
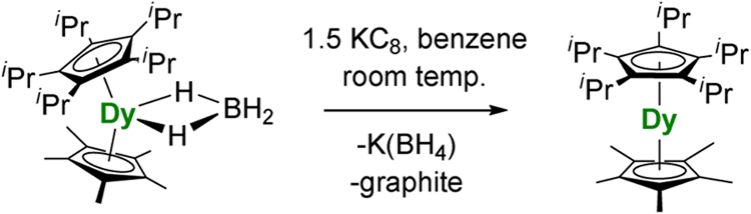
Synthesis of [(η^5^-C_5_^*i*^Pr_5_)­Dy­(η^5^-Cp*)]

### Solid-State Structure

The molecular structure of [(η^5^-C_5_^*i*^Pr_5_)­Dy­(η^5^-Cp*)] was determined by single-crystal X-ray diffraction and is shown in Figure 1. Molecules of the complex crystallize in the triclinic space group *P*1̅ with 0.5 hexane molecules per formula unit (Table S1). The dysprosium­(II) center is sandwiched between the η^5^-C_5_^*i*^Pr_5_ and η^5^-Cp* ligands to give a linear sandwich complex with a Cp_cent_-Dy-Cp_cent_ angle of 179.643(9)° (“cent” denotes the centroid of the ligand) and a dihedral angle between the mean planes of the two ligands of 0.39(19)°, reflecting their mutually parallel orientation. The angle subtended at dysprosium in this complex is much wider than the analogous angle of 162.507(1)° in the dysprosium­(III) cation [(η^5^-C_5_^*i*^Pr_5_)­Dy­(η^5^-Cp*)]^+^.^19^ The C_5_^*i*^Pr_5_ ligand in the divalent dysprosocene is disordered and was refined assuming that the isopropyl groups are disordered over two opposite orientations. The dysprosium center does not exhibit any disorder. The Dy–C bond lengths to the C_5_^*i*^Pr_5_ and Cp* ligands are in the range 2.638(4)-2.657(4) Å and 2.608(5)-2.639(4) Å, respectively, and the Dy–(C_5_^*i*^Pr_5_)_cent_ and Dy–(Cp*)_cent_ distances are 2.3587(3) Å and 2.3376(3) Å, respectively (Table S2). Compared to [(η^5^-C_5_^*i*^Pr_5_)­Dy­(η^5^-Cp*)]^+^, which has Dy–(C_5_^*i*^Pr_5_)_cent_ and Dy–(Cp*)_cent_ distances of 2.284(1) Å and 2.296(1) Å, respectively,^19^ a slight lengthening of the metal ligand distances by 0.042–0.075 Å occurs upon reduction. Previous studies have shown that changes in the metal-Cp distances when trivalent rare-earth metals are reduced to their divalent form can indicate whether the electronic configuration of the lower oxidation state is 4f^*n*^5d^1^ or 4f^*n*+1^.^38^ The structural changes observed for [(η^5^-C_5_^*i*^Pr_5_)­Dy­(η^5^-Cp*)] support a 4f*^n^* 5d^1^ configuration. Furthermore, the Dy-(C_5_^*i*^Pr_5_)_cent_ and Dy-(Cp*)_cent_ distances in [(η^5^-C_5_^*i*^Pr_5_)­Dy­(η^5^-Cp*)] are shorter by 0.026 Å and 0.047 Å, respectively, than the analogous distance of 2.385(1) Å in [(η^5^-C_5_^*i*^Pr_5_)_2_Dy], presumably due to the smaller size of Cp*. The shortest intermolecular Dy···Dy distance in the structure of [(η^5^-C_5_^*i*^Pr_5_)­Dy­(η^5^-Cp*)] is 8.9820(9) Å.

**Figure 1:**
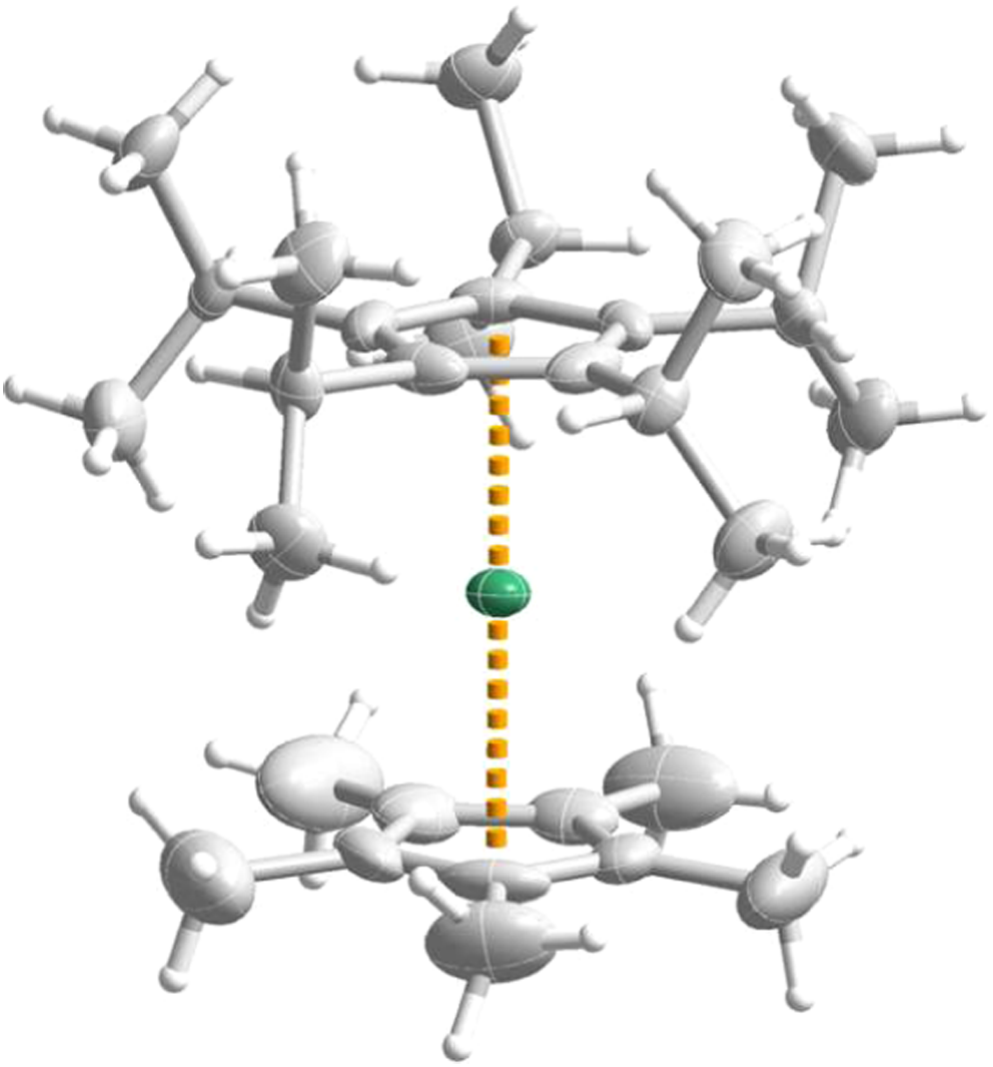
Molecular structure of [(η^5^-C_5_^*i*^Pr_5_)­Dy­(η^5^-Cp*)] with thermal ellipsoids at the 50% probability level. C: gray; H: white; Dy: green.

### UV/Vis/NIR Spectroscopy

UV/vis/NIR absorption spectra in the range 200–1000 nm were recorded on hexane solutions of the precursor [(η^5^-C_5_^*i*^Pr_5_)­Dy­(η^5^-Cp*)­(BH_4_)] and [(η^5^-C_5_^*i*^Pr_5_)­Dy­(η^5^-Cp*)] (Figures 2 and S3–S11). Both compounds produce similar absorptions in the high-energy region around 220, 280, and 340 nm. The almost colorless solution of the trivalent dysprosium borohydride precursor is reflected in the absence of any significant absorptions in the visible region. In contrast, hexane solutions of [(η^5^-C_5_^*i*^Pr_5_)­Dy­(η^5^-Cp*)] are orange, consistent with the strong absorption at 472 nm in the visible region. This distinct UV/vis/NIR spectra characteristic is comparable to that of previously reported [(η^5^-C_5_^*i*^Pr_5_)_2_Dy], which was also found to have a 4f^9^5d^1^ electron configuration.^48^

**Figure 2:**
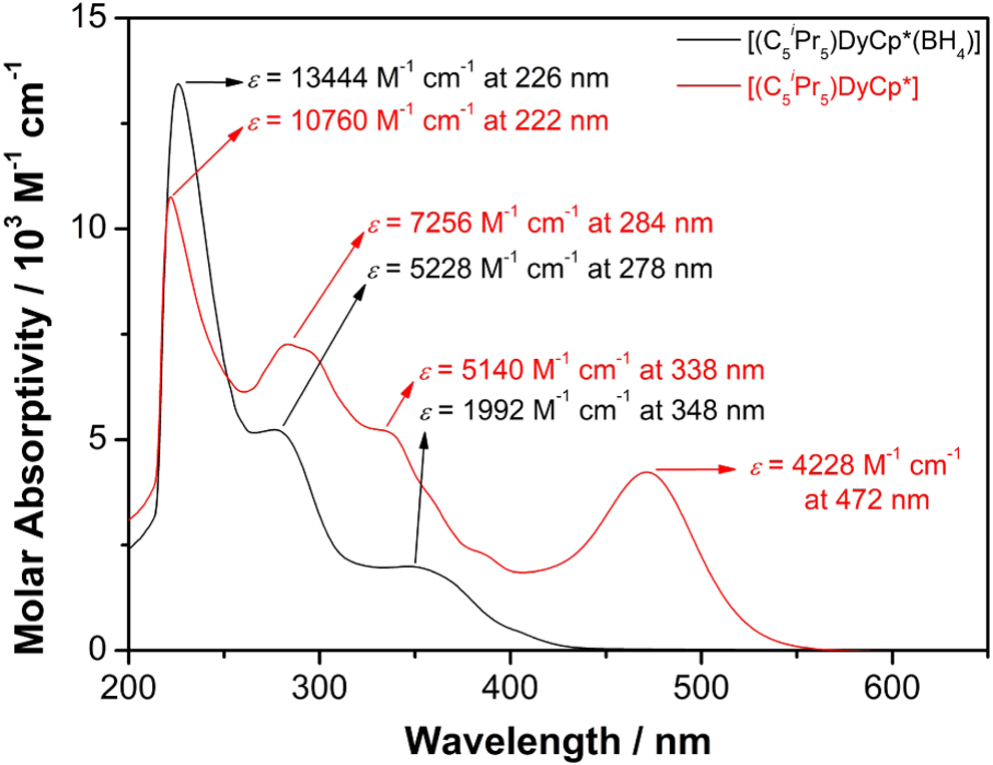
UV/vis/NIR spectrum of [(η^5^-C_5_^*i*^Pr_5_)­Dy­(η^5^-Cp*)­(BH_4_)] (in black) and [(η^5^-C_5_^*i*^Pr_5_)­Dy­(η^5^-Cp*)] (in red) in hexane at room temperature.

Interpretation of the UV/vis/NIR spectrum of [(η^5^-C_5_^*i*^Pr_5_)­Dy­(η^5^-Cp*)] was aided with the use of time-dependent density functional theory (TD-DFT) calculations (Figure S33). The calculations utilized a 4f-in-core effective core potential to avoid issues arising from the strong electron correlation effects within the 4f shell. Full computational details are provided in the Supporting Information. Consequently, the predicted transitions do not take the role of the 4f electrons into account. The orbitals involved in the transitions are depicted in Figure 6 and discussed in detail in the Theoretical Calculations section.

The peaks observed at 222, 284 and 338 nm in the UV/vis/NIR spectrum of [(η^5^-C_5_^*i*^Pr_5_)­Dy­(η^5^-Cp*)] were reasonably well reproduced by sets of transitions calculated to occur in the regions 218–225, 282–286 nm and 306–309 nm (Table S6). An additional set of peaks was predicted to occur at 389–392 nm, most likely corresponding to the experimentally observed peak at 472 nm. Therefore, the three shortest wavelength peaks can be reliably interpreted. The 222 nm peak corresponds to a transition from a dysprosium 6s/5d orbital to a 5d orbital with π* character. The 284 nm peak corresponds to ligand-to-metal charge-transfer excitations from the occupied cyclopentadienyl π-orbitals to a vacant dysprosium 5d orbital with δ-character, and the 338 nm peak corresponds to ligand-to-metal charge-transfer excitations from the occupied cyclopentadiene π orbitals to the dysprosium 6s/5d orbital. The peaks predicted at 389–392 nm, and probably observed experimentally at lower energy, are tentatively assigned to excitations from the 6s/5d orbital to diffuse metal or ligand orbitals.

To test the stability of [(η^5^-C_5_^*i*^Pr_5_)­Dy­(η^5^-Cp*)] in solution, UV/vis/NIR spectra (Figure S12) on the same sample were collected at various intervals over 12 h. The shape and intensity of the absorptions are virtually unchanged over 12 h. Indeed, the orange color of hexane solutions of [(η^5^-C_5_^*i*^Pr_5_)­Dy­(η^5^-Cp*)] does not change for up to a month at room temperature under an inert atmosphere, reflecting the good stability of the divalent compound in solution over time. Similar stability was found in benzene solution, but in THF the orange color quickly dissipates due to decomposition.

### Static Magnetic Properties

Variable-temperature direct current (DC) magnetic susceptibility data were collected upon warming the sample from 100 to 300 K in an applied field of 1000 Oe (Figure 3). The sample was restrained in Fomblin oil since [(η^5^-C_5_^*i*^Pr_5_)­Dy­(η^5^-Cp*)] dissolves in the more common medium eicosane upon melting. The χ_M_ *T* curve exhibits a distinct step near the pour point (238 K) of the oil, indicating crystal torque effects in the applied magnetic field. Therefore, with an abundance of caution, we only discuss data below 217 K. The χ_M_ *T* value (where χ_M_ is the molar magnetic susceptibility) is 15.40 cm^3^ K mol^–1^ at 100 K, which increases slowly as the temperature rises, reaching 16.20 cm^3^ K mol^–1^ at 217 K. To the best of our knowledge, the χ_M_ *T* value at 217 K is the largest observed for a dysprosium­(II) compound, even when compared to compounds measured at 300 K, and equates to an effective magnetic moment of 11.38 μ_B_. The μ_eff_ value for [(η^5^-C_5_^*i*^Pr_5_)­Dy­(η^5^-Cp*)] at 217 K is equal to the highest μ_eff_ value in a mononuclear complex, which was reported for the divalent holmium complex [Ho­{C_5_H_4_(SiMe_3_)}_3_]^−^ at 300 K.^45^ The room temperature value of χ_M_ *T* and μ_eff_ for [(η^5^-C_5_^*i*^Pr_5_)­Dy­(η^5^-Cp*)] should, of course, be somewhat larger. The χ_M_ *T* value is also significantly higher than the theoretical value of 14.07 cm^3^ K mol^–1^ for a 4f^10^ ion (*S* = 2, *L* = 6, *J* = 8, *g*_*J*_ = 5/4, 5/8),^56^ indicating that the electron configuration of [(η^5^-C_5_^*i*^Pr_5_)­Dy­(η^5^-Cp*)] should be 4f^9^5d^1^.

**Figure 3:**
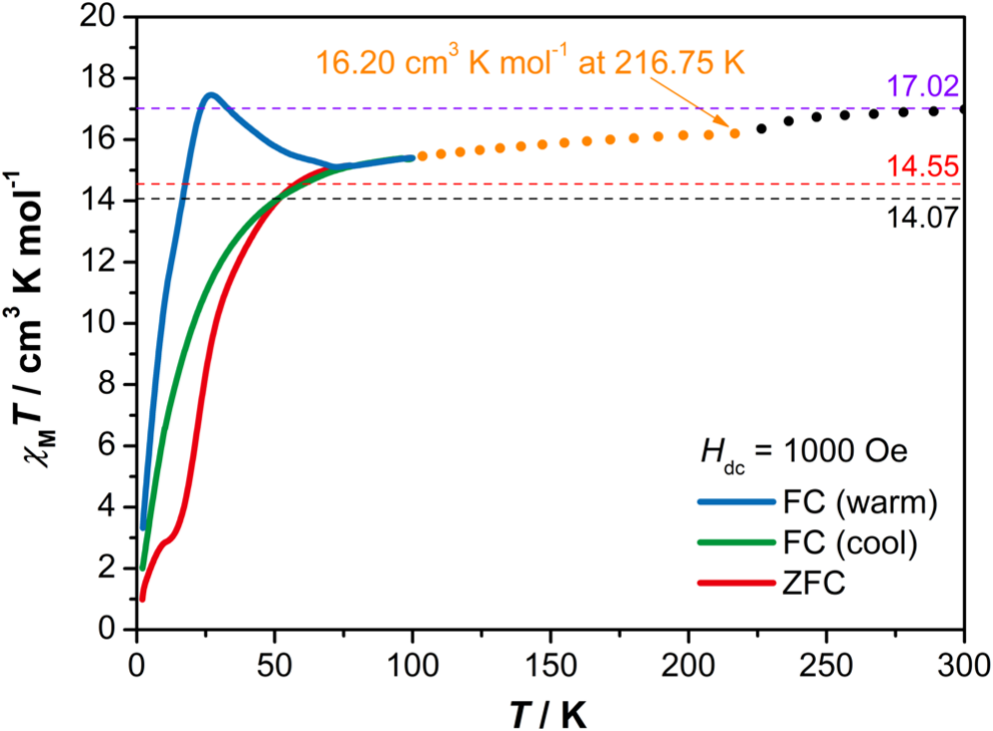
Temperature-dependent DC magnetic susceptibility data for [(η^5^-C_5_^*i*^Pr_5_)­Dy­(η^5^-Cp*)]·0.5hexane. The black dashed line corresponds to a χ_M_ *T* value of 14.07 cm^3^ K mol^–1^ for a 4f^10^ configuration, and the red dashed line corresponds to a χ_M_ *T* value of 14.55 cm^3^ K mol^–1^ for an uncoupled 4f^9^5d^1^ configuration. The violet dashed line corresponds to a χ_M_ *T* value of 17.02 cm^3^ K mol^–1^ for the coupled 4f^9^5d^1^ configuration.

In previous studies, Cloke et al. introduced three limiting cases to describe the interactions between 4f electrons and additional electrons in zerovalent rare-earth bis­(arene) sandwich compounds,^57–59^ an idea later extended to the divalent rare-earth metallocenes by Long et al.^48,60^ For a divalent dysprosium compound with 4f^9^5d^1^ configuration, the first limiting case is when the spin–spin coupling between the 5d and 4f electrons is less than *k*_B_ *T* (*k*_B_ is the Boltzmann constant); in this case, the expected χ_M_ *T* value of 14.55 cm^3^ K mol^–1^ is the sum of the expected values for a Dy^3+^ ion (14.17 cm^3^ K mol^–1^) and a free electron (0.375 cm^3^ K mol^–1^). The other two limiting cases consider the spin–spin coupling to be greater than *k*_B_ *T*, one case where the spin–spin coupling is much greater than the 4f spin–orbit coupling (SOC), and the other where the spin–spin coupling is much less than the 4f SOC. In the latter two cases, the χ_M_ *T* value is the same (17.02 cm^3^ K mol^–1^) for the 4f^9^5d^1^ configuration, regardless of the relative magnitude of the two types of coupling. For [(η^5^-C_5_^*i*^Pr_5_)­Dy­(η^5^-Cp*)], the χ_M_ *T* value of 16.20 cm^3^ K mol^–1^ at 217 K is more consistent with the latter two situations where *k*_B_ *T* is not dominant, making χ_M_ *T* for our divalent dysprosocene comparable to that of 16.1 cm^3^ K mol^–1^ reported for [K­(2,2,2-crypt)]­[Dy­(Cp′)_3_].^45^ In contrast, [(η^5^-C_5_^*i*^Pr_5_)_2_Dy] exhibits a χ_M_ *T* value of 15.15 cm^3^ K mol^–1^ at 300 K, which is thought to be more in line with the first coupling regime.

To identify magnetic blocking in [(η^5^-C_5_^*i*^Pr_5_)­Dy­(η^5^-Cp*)], field-cooled (FC) and zero-field-cooled (ZFC) variable-temperature (2 K min^–1^) magnetic susceptibility data were collected under an applied field of 1000 Oe in the range of 2–100 K (Figures 3 and S13). The bifurcation observed between the FC (warming and cooling modes) and ZFC curves at approximately 70 K suggests prominent magnetic blocking.

### Dynamic Magnetic Properties

In zero DC field, the magnetization dynamics of [(η^5^-C_5_^*i*^Pr_5_)­Dy­(η^5^-Cp*)] were initially investigated by measuring the real and imaginary components of the AC susceptibility (χ′ and χ″, respectively) as functions of temperature at six AC frequencies (ν) in the range 1–999 Hz. Two sets of maxima were observed, corresponding to a fast relaxation process below 17.6 K and a much slower relaxation process observable up to 106 K (Figures S14–S15). These observations imply a complicated relaxation situation in a compound consisting of a single dysprosium­(II) ion. Other examples of mononuclear SMMs show similar behavior, including [(η^5^-C_5_^*i*^Pr_5_)_2_M] with M = Tb and Dy, both of which also do not exhibit crystallographically disordered metal centers.^48^ For [(η^5^-C_5_^*i*^Pr_5_)­Dy­(η^5^-Cp*)], the stability of the compounds has been established, and likewise the phase purity was verified through unit cell measurements of multiple single crystals. The results of magnetic measurements are reproducible across four different batches of independently synthesized samples.

Detailed measurements of χ″(ν) in the high temperature regime show well-defined maxima in the range 74–106 K and frequencies of ν = 0.1–999 Hz (Figures 4 and S16–S18). The magnetic relaxation time (τ) were extracted from the Cole–Cole plots of χ″ versus χ′ (Table S3 and Figures S19–S20) using the generalized Debye model, which was fitted with α-parameters of 0.03–0.20, revealing a narrow relaxation time distribution. At 74 K, the high-temperature relaxation process has slowed down to the point where it cannot be measured further using AC susceptibility, hence magnetization decay experiments were employed to investigate the magnetic relaxation behavior at lower temperatures (Figures S21–S24 and Table S4). Fitting the plots of the remnant magnetization versus time yields the relaxation rates. The temperature-dependent relaxation time combined from AC and DC measurements (Figures S25) was fitted by the equation τ^–1^ = τ_0_^–1^ e^–*U*_eff_/*k*_B_ *T*^ + *CT* ^*n*^ + τ_QTM_^–1^ (τ_0_, *U*_eff_, *C* and *n*, are the attempt time, effective spin-reversal barrier, Raman coefficient and Raman exponent, respectively, and τ_QTM_^–1^ is the rate of quantum tunneling of the magnetization). This analysis gives *U*_eff_ = 1551 cm^–1^, τ_0_ = 1.2 × 10^–13^ s, *C* = 1.8 × 10^–6^ s^–1^ K^–*n*^ *n* = 2.0 and τ_QTM_ = 4504 s. The temperature dependent relaxation time in the faster, low-temperature process (Figure S26) was fitted using only the Raman expression τ^–1^ = *CT* ^*n*^, giving *C* = 9.3 × 10^–3^ s^–1^ K^–*n*^ and *n* = 4.7. Based on the magnetization decay measurements, a 100 s blocking temperature of 62 K applies to [(η^5^-C_5_^*i*^Pr_5_)­Dy­(η^5^-Cp*)].

**Figure 4:**
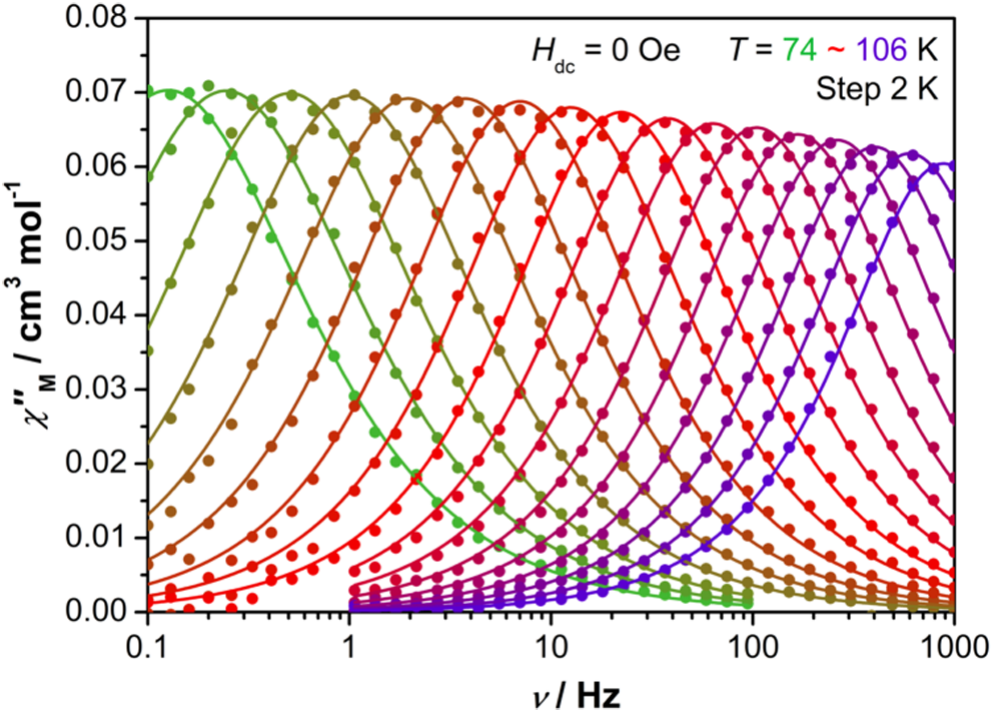
Plot of χ″(ν) for [(η^5^-C_5_^*i*^Pr_5_)­Dy­(η^5^-Cp*)]·0.5hexane in zero DC field at AC frequencies of 0.1–999 Hz from 74 to 106 K. Solid lines represent fits to the data using a generalized Debye equation.

The large *U*_eff_ value of 1551 cm^–1^ associated with the Orbach relaxation in [(η^5^-C_5_^*i*^Pr_5_)­Dy­(η^5^-Cp*)] is remarkably similar to that of 1541 cm^–1^ determined for [(η^5^-C_5_^*i*^Pr_5_)­Dy­(η^5^-Cp*)]­[B­(C_6_F_5_)_4_],^19^ placing the divalent dysprosocene among the best performing SMMs in terms of effective energy barrier. The energy barrier for [(η^5^-C_5_^*i*^Pr_5_)­Dy­(η^5^-Cp*)] is also the highest measured for a divalent lanthanide SMM, exceeding the previous record of 1365 cm^–1^ reported for [Dy­(Piso)_2_] by approximately 14%.^50^ Furthermore, the contrast between the AC susceptibility properties of [(η^5^-C_5_^*i*^Pr_5_)­Dy­(η^5^-Cp*)] and those of the structurally similar compound [(η^5^-C_5_^*i*^Pr_5_)_2_Dy], which shows two relaxation processes but no obvious Orbach process,^48^ could hardly be starker.

### Magnetic Hysteresis

Considering that open magnetization (*M*) versus field (*H*) hysteresis loops are important considerations for the development of data storage applications, the variable-field magnetization properties of [(η^5^-C_5_^*i*^Pr_5_)­Dy­(η^5^-Cp*)] were measured. Using a field sweep rate of 200 Oe s^–1^, open *M*(*H*) hysteresis loops were observed at 2 K with a coercive field of *H*_c_ = 31085 Oe and a remanent magnetization of *M*_r_ = 2.30 Nβ. Under the maximum field of +70 kOe at 2 K, the magnetization saturates at 5.36 Nβ (Figure 5). Reducing the field results in a slow decrease in the magnetization accompanied by step-like drops at +9183, +364, −2536, and −8237 Oe (Figure S27), possibly indicating hyperfine-driven QTM due to coupling with the nuclear spins of ^161^Dy and ^163^Dy (19 and 25% abundance, respectively, both *I* = 5/2 nuclei).^61–63^ Increasing the field in the opposite direction from −10 to – 70 kOe produces an almost linear decrease in the magnetization, with a value of −1.93 Nβ reached at the maximum field of 70 kOe, where the hysteresis loops remain open.

**Figure 5:**
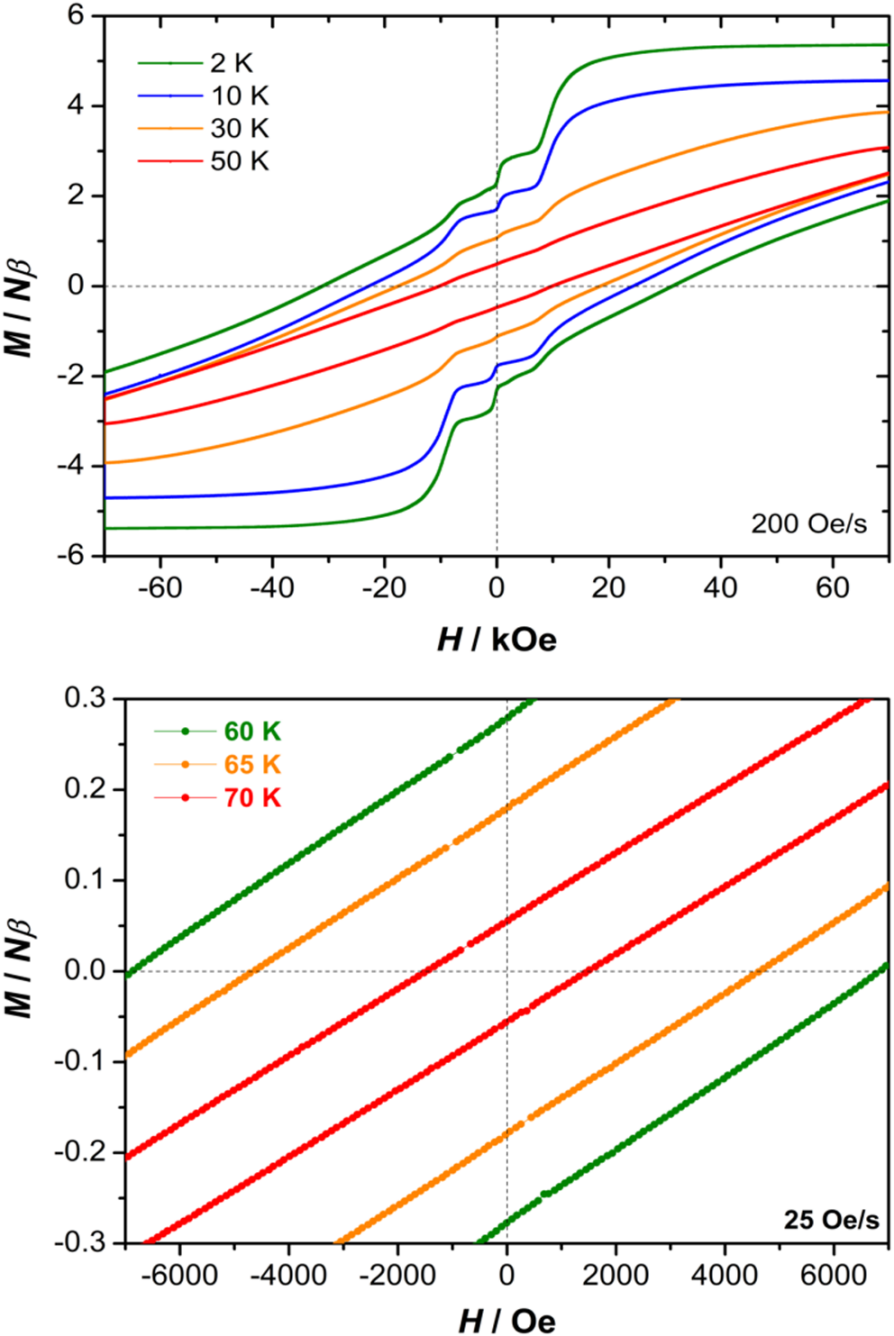
Magnetic hysteresis loops for [(η^5^-C_5_^*i*^Pr_5_)­Dy­(η^5^-Cp*)]·0.5hexane. Upper: data collected continuously at 2, 10, 30, and 50 K using a field sweep speed of 200 Oe s^–1^. Lower: data collected continuously at 60, 65, and 70 K using a field sweep speed of 25 Oe s^–1^.

The hysteresis loops gradually close with increasing temperature, with *H*_c_ = 10343 Oe and *M*_r_ = 0.5 Nβ at 50 K. Even at this temperature, however, the opening of the *M*(*H*) loop is still quite pronounced, indicative of hard magnet-like behavior in [(η^5^-C_5_^*i*^Pr_5_)­Dy­(η^5^-Cp*)]. With further increases in temperature, the coercivity and remanent magnetization drop to *H*_c_ = 225 Oe and *M*_r_ = 0.01 Nβ at 75 K, and with the loops being virtually closed at 80 K, with *H*_c_ = 25 Oe and *M*_r_ = 0.001 Nβ (Figures 5, S28–S31, and Table S5).

Using a low field sweep rate of 25 Oe s^–1^, the coercive fields at 60, 65, and 70 K are 6875, 4655, and 1481 Oe, respectively (Figures 5 and S32), indicating that the hysteresis-derived blocking temperature should be 70 K, in good agreement with the FC-ZFC susceptibility. Intriguingly, the linear dysprosocene [(η^5^-C_5_^*i*^Pr_5_)_2_Dy] displays butterfly hysteresis loops up to 75 K that close around zero field, even though Orbach relaxation does not occur around this temperature.^48^ In contrast, [Dy­(Piso)_2_] has an energy barrier of 1365 cm^–1^, but again its hysteresis is butterfly shaped and the loops already closed at zero field by 2 K.^50^ Consequently, [(η^5^-C_5_^*i*^Pr_5_)­Dy­(η^5^-Cp*)] appears to be an unusual dysprosium­(II) SMM by virtue of its high effective energy barrier and open hysteresis loops close to the boiling point of liquid nitrogen.

### Theoretical Calculations

The electronic structure of [(η^5^-C_5_^*i*^Pr_5_)­Dy­(η^5^-Cp*)] was studied at NEVPT2//SA-CASSCF level of theory using the ORCA code version 5.0.4.^64–70^ SOC was treated using the well-established quasi-degenerate perturbation theory (QDPT) approach.^71,72^ The active space consisted of 14 electrons in 14 orbitals. The active orbitals are shown in Figure 6 and consist of the seven 4f orbitals, bonding and antibonding combinations of cyclopentadienyl orbitals and the π-symmetric 5d orbitals (denoted as 5d_π_ and 5d_π_*, respectively), a toroidal 6s/5d orbital combination and nonbonding δ-symmetric 5d orbitals (denoted as 5d_δ_). The ground electronic configuration is calculated to be 4f^9^ (6s/5d)^1^, consistent with the DC magnetic susceptibility measurements and similar to previously reported calculations on [(η^5^-C_5_^*i*^Pr_5_)_2_Dy].^48^ The mixed 6s/5d orbital in [(η^5^-C_5_^*i*^Pr_5_)­Dy­(η^5^-Cp*)] consists of 22% 6s, 52% 5d, and minor contributions from other orbitals, and is reminiscent of the electronic configuration of [(η^5^-C_5_^*i*^Pr_5_)_2_U], which features a (7s/6d)^1^ highest-occupied molecular orbital (HOMO).^73,74^

**Figure 6:**
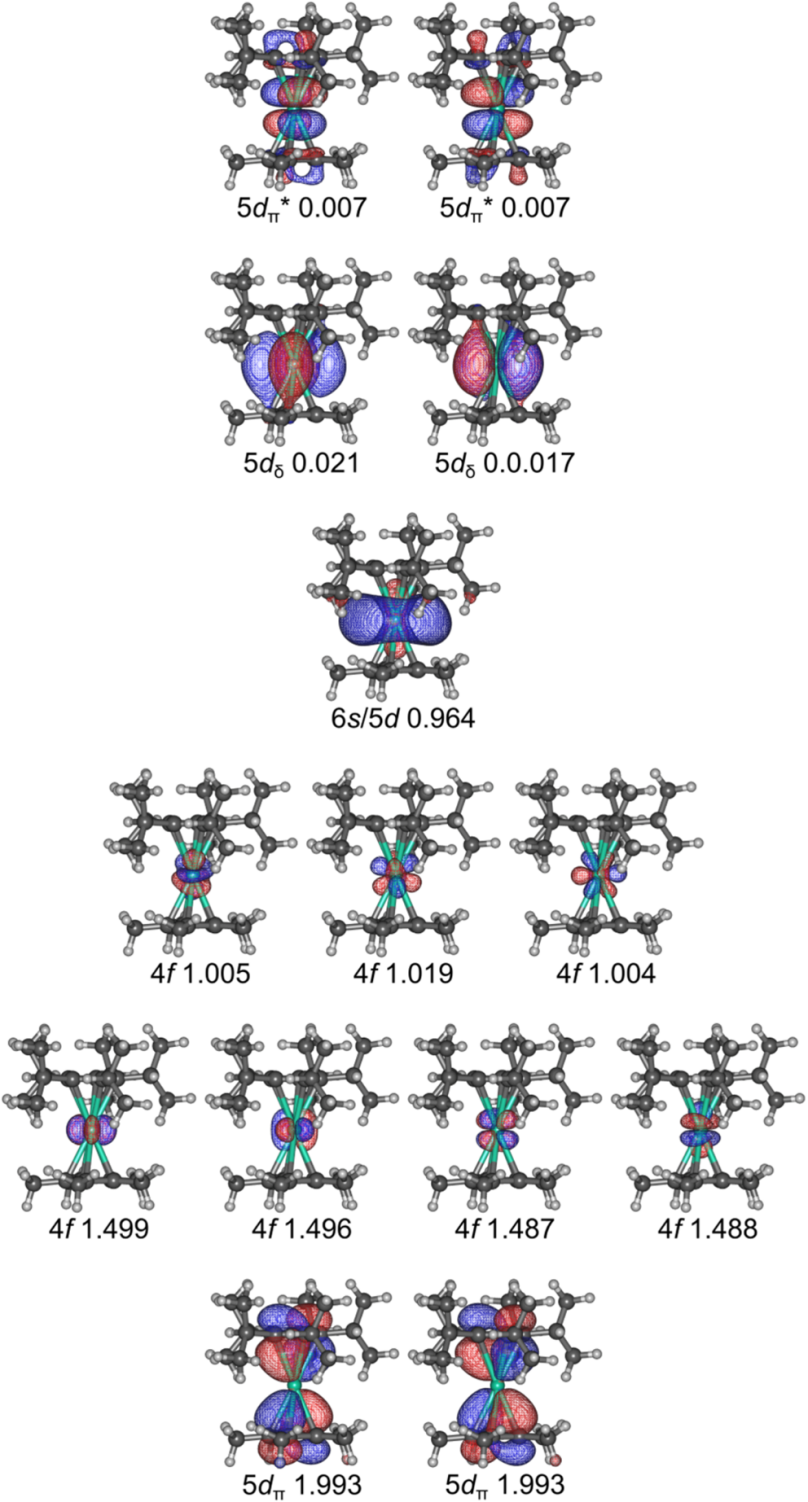
Natural SA-CASSCF orbitals calculated for [(η^5^-C_5_^*i*^Pr_5_)­Dy­(η^5^-Cp*)]. The numbers indicate the orbital occupations in the ground spin-state.

Before the inclusion of SOC in [(η^5^-C_5_^*i*^Pr_5_)­Dy­(η^5^-Cp*)], the lowest 54 septet and 54 quintet spin states form a manifold of states up to 15,000 cm^–1^ that is clearly separate from the higher-lying manifolds of states (Table S9). By inspection of orbital occupations and splitting patterns, these sets of states can be associated with the states that arise from the coupling of the ^6^H and ^6^F terms in [(η^5^-C_5_^*i*^Pr_5_)­Dy­(η^5^-Cp*)] arising from the 4f^9^ configuration with a single additional electron in a (6s/5d)^1^ or 5d_δ_^1^ configuration. The lowest spin-quintet state lies at 3529 cm^–1^ above the ground spin-septet state. In a simple isotropic exchange coupling model, this would indicate a ferromagnetic Hund’s rule exchange-coupling parameter of 3529 cm^–1^ between the 4f^9^ spin and the (6s/5d)^1^ spin. This parameter is comparable to the splitting between different *J*-multiplets in a Dy^3+^ ion and means that the electronic structure will most likely deviate from what would be expected in the case of ideal *L*–*S* coupling. Upon the inclusion of SOC, the spin-septet and spin-quintet manifolds in [(η^5^-C_5_^*i*^Pr_5_)­Dy­(η^5^-Cp*)] become strongly mixed, with no discernible manifolds corresponding to *J*-multiplet structures (Table S10).

Due to the even number of electrons, [(η^5^-C_5_^*i*^Pr_5_)­Dy­(η^5^-Cp*)] is a non-Kramers system. The lowest-energy spin–orbit coupled states are nearly degenerate quasi-doublets. The ground quasi-doublet and the first-excited doublet are exactly degenerate up to the numerical accuracy of the calculations. The second-excited doublet has a splitting of less than 0.1 cm^–1^ and the third excited doublet has a splitting of 7 cm^–1^, with the splitting increasing in the higher-lying states. Quasi-doublets are exactly degenerate in axial symmetry. An axial symmetry is not broken by the occupation of the 6s/5d orbital in [(η^5^-C_5_^*i*^Pr_5_)­Dy­(η^5^-Cp*)] as this orbital consists of the 6s and 5d_*z* ^2^_ orbitals, and both of these interact in exactly the same manner with pairs of 4f orbitals with one-particle orbital angular momentum values ±*m_l_*. Occupation of the 5d_*z* ^2^_ orbitals would lead to orbital degeneracy that would most likely be lifted by Jahn–Teller distortions.^75^ However, the 6s/5d orbital is nondegenerate and its toroidal shape most likely promotes a more linear Cp–Dy–Cp angle, reducing transverse components of the crystal field (CF) that affect the 4f state. However, it is not immediately clear whether the 6s/5d electron in [(η^5^-C_5_^*i*^Pr_5_)­Dy­(η^5^-Cp*)] would stabilize the doublets with the largest absolute values of ±(*M*_*J*_ + *m*_*s*_), where *M*_*J*_ is the total angular momentum projection of the ground 4f^9^ multiplet and *m*_*s*_ is the one-particle spin projection of the 6s/5*d* electron, or if it would stabilize those with the smallest or some intermediate values of ±(*M*_*J*_ + *m*_*s*_).

To better understand the magnetic nature of the lower-energy quasi-doublets in [(η^5^-C_5_^*i*^Pr_5_)­Dy­(η^5^-Cp*)], the **g-**tensors corresponding to pseudospin describing the doublets were calculated using the SINGLE_ANISO module.^76^ The results are listed in Table S7. Griffith’s theorem^77^ posits that the transverse components of the **g**-tensors are zero and only the axial component has a nonzero value in a non-Kramers system. In an ideally axial doublet corresponding to the maximal angular-momentum projections ±(*M*_*J*_ + *m*_*s*_) = ±(*J* + *s*) = ±8, the axial component of the ***g***-tensor should be *g*_*z*_ = 22. This calculation assumes *L–S* coupling and that the Landé *g*-factor of the 4f^9^ *J*-multiplet is *g*_*J*_ = 4/3, with a free-electron *g*-factor of *g*_e_ = 2.^78^ The principal component of the **g**-tensor calculated for the ground quasi doublet is *g*_*z*_ = 21.979, which is very close to the ideal axial case. The small deviation from *g*_*z*_ = 22 most likely results from mixing of different *L–S* terms by the 4f^9^-(6s/5d)^1^ Hund’s rule exchange.

The principal magnetic axis of the ground quasi-doublet in [(η^5^-C_5_^*i*^Pr_5_)­Dy­(η^5^-Cp*)] is shown in Figure 7 and closely follows the principal molecular pseudosymmetry axis. The magnetic axiality of the higher-lying quasi-doublets gradually decreases with increasing energy.

**Figure 7:**
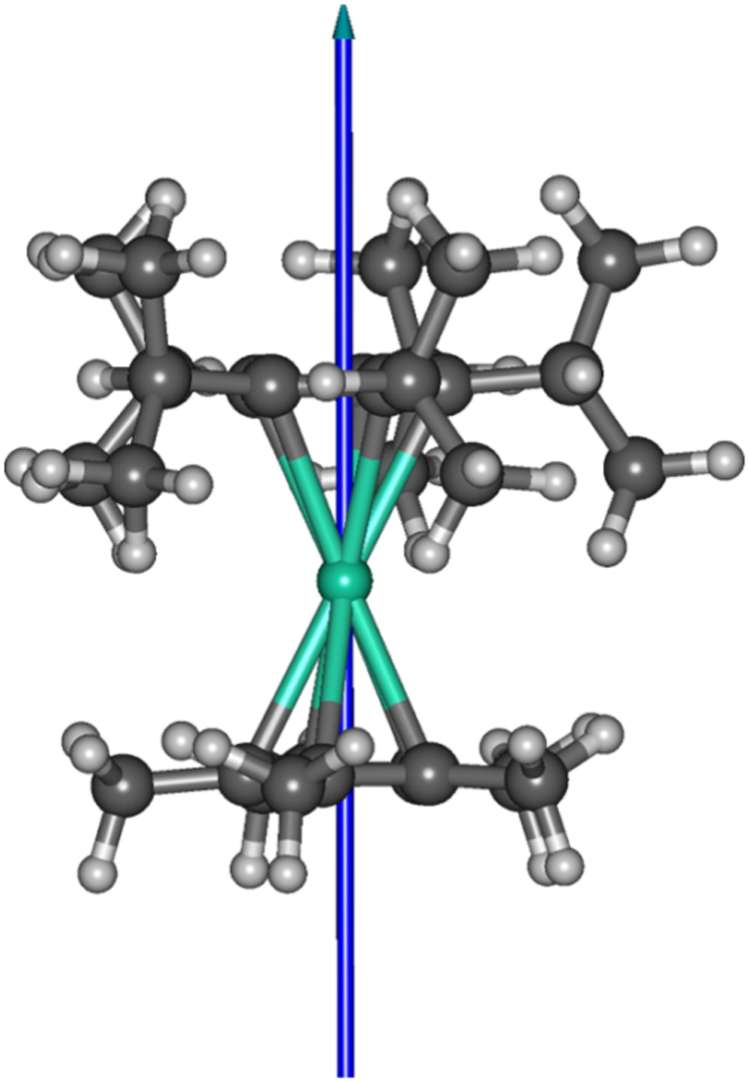
Principal magnetic axis of the ground quasi-doublet calculated for [(η^5^-C_5_^*i*^Pr_5_)­Dy­(η^5^-Cp*)].

The near-perfect degeneracy and high axiality of the ground quasi doublet indicate that [(η^5^-C_5_^*i*^Pr_5_)­Dy­(η^5^-Cp*)] should be a high-performance SMM, as observed experimentally. To study the magnetic relaxation mechanism, a qualitative effective barrier was calculated using the well-established phenomenological methodology where the relaxation pathway is traced by following the largest transition magnetic moment matrix elements between different states (Table S8).^79^ The barrier is shown in Figure 8. The transition magnetic moments predict that the Orbach mechanism should involve at least the third excited quasi-doublet at 2288 cm^–1^. However, this is considerably higher than the experimental barrier of 1551 cm^–1^. The experimental *U*_eff_ value is between the first and second excited doublets at 888 and 1733 cm^–1^, respectively. While ‘underbarrier’ relaxation has been previously observed in SMMs,^36,80^ the most likely reason for the discrepancy is that the calculations underestimate the electron correlation effects between the 6s/5d orbital and the ligands that is only included up to second order in perturbation theory. Including the missing electron correlation in the calculations on [(η^5^-C_5_^*i*^Pr_5_)­Dy­(η^5^-Cp*)] would increase the effects of the lower-symmetry components of the crystal-field on the 6s/5d orbital, which should reduce the axiality of the higher-lying quasi-doublets. This would lower the energy of the doublets, and the transition magnetic moments would increase, hence relaxation via the second-excited doublet would be plausible. Unfortunately, the electron correlation effects cannot feasibly be accounted for beyond the level of theory already used due to the rapidly increasing computational costs.

**Figure 8:**
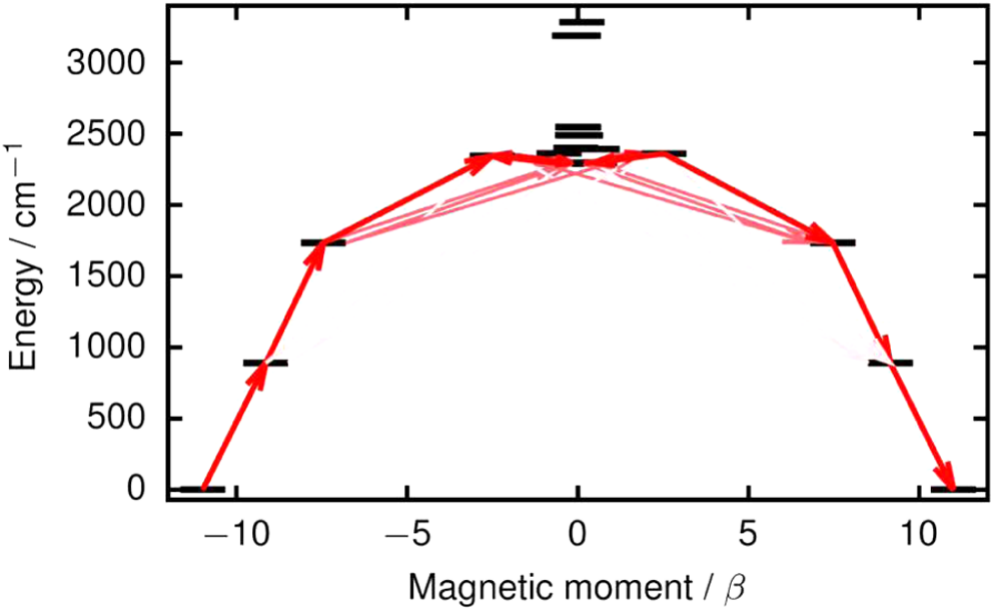
Qualitative effective barrier for the relaxation of magnetization in [(η^5^-C_5_^*i*^Pr_5_)­Dy­(η^5^-Cp*)]. Thicker arrows indicate stronger transition magnetic moment matrix elements between the respective states. Transitions involving higher excited states are omitted for clarity.

Considering the magnetic properties of [(η^5^-C_5_^*i*^Pr_5_)­Dy­(η^5^-Cp*)] in light of the computational results, the emerging design criteria for a high-performance dysprosium­(II) SMM do not appear to be substantially different to those required for dysprosium­(III), at least with metallocene-like structures. Our results show that a strong axial crystal field combined with a negligible equatorial crystal field are responsible for the large *U*_eff_ value and the open hysteresis loops at relatively high temperatures. The SMM properties of [(η^5^-C_5_^*i*^Pr_5_)­Dy­(η^5^-Cp*)] very likely benefit substantially from the near-perfect linear geometry of the sandwich complex. These suggestions are broadly in agreement with conclusions drawn on the divalent dysprosium SMM [M­(Piso)_2_], which has a 4f^9^5d^1^ configuration and shows a high *U*_eff_ value of 1365 cm^–1^ but with no open hysteresis loops, consistent with a transverse contribution to the crystal field from the off-axis amidinate *N*-donor atoms.^50^ Consequently, slight distortions away from ideal linearity may not impact dramatically on *U*_eff_ but seemingly do affect the hysteresis properties, as is usually observed with dysprosium­(III) SMMs. However, given that [(η^5^-C_5_^*i*^Pr_5_)­Dy­(η^5^-Cp*)] and [(η^5^-C_5_^*i*^Pr_5_)_2_Dy] are isolobal, isoelectronic, essentially isostructural and have similar ligand rigidity, the contrast in their SMM properties is hard to explain and remains an open question. If anything, these two metallocenes highlight that the electronic structure and dynamic magnetism of dysprosium­(II) is nontrivial.

## Conclusions

Reduction of [(η^5^-C_5_^*i*^Pr_5_)­Dy­(η^5^-Cp*)­(BH_4_)] with KC_8_ produces the divalent dysprosocene [(η^5^-C_5_^*i*^Pr_5_)­Dy­(η^5^-Cp*)] in 40% yield. Structural studies reveal a near-perfect linear geometry for the dysprosocene. Lengthening of the dysprosium-centroid distances to the cyclopentadienyl ligands relative to the dysprosocenium cation [(η^5^-C_5_^*i*^Pr_5_)­Dy­(η^5^-Cp*)]^+^ suggest that the divalent dysprosium center adopts a 4f^9^5d^1^ configuration. DC magnetic susceptibility measurements reveal that χ_M_ *T* for [(η^5^-C_5_^*i*^Pr_5_)­Dy­(η^5^-Cp*)] at 217 K is 16.20 cm^3^ K mol^–1^, suggesting strong coupling of the 4f and 5d electrons. The resulting effective magnetic moment at 217 K is 11.38 μ_B_, equaling the highest μ_eff_ yet recorded for a mononuclear complex. Combined AC susceptibility and magnetization decay experiments established the SMM behavior of [(η^5^-C_5_^*i*^Pr_5_)­Dy­(η^5^-Cp*)], which consists of high-temperature Orbach relaxation up to 106 K characterized by an energy barrier of *U*_eff_ = 1551 cm^–1^, a record for a dysprosium­(II) SMM. A 100-s blocking temperature of 62 K was also determined from magnetization decay experiments. Hard magnet properties were established for [(η^5^-C_5_^*i*^Pr_5_)­Dy­(η^5^-Cp*)] using hysteresis measurements from 2 to 80 K, with the *M*(*H*) loops remaining open at 70 K with a scan rate of 25 Oe s^–1^. Analysis of [(η^5^-C_5_^*i*^Pr_5_)­Dy­(η^5^-Cp*)] using multireference calculations revealed that the Dy^2+^ center occupies a coordination environment that is as close to perfectly axial as can reasonably be expected for a heteroleptic complex. Although departures from the *L*–*S* coupling model render calculations on [(η^5^-C_5_^*i*^Pr_5_)­Dy­(η^5^-Cp*)] challenging, the dominant relaxation in this SMM probably proceeds via the second-excited doublet.

While the development of design criteria for divalent dysprosium SMMs is a work in progress given the very small number of examples, similarities with trivalent dysprosium SMMs are evident, especially the requirement for strong axial crystal fields to underpin large *U*_eff_ values and hysteresis with coercivity. Our ongoing work will explore the possibilities in greater depth.

## Supplementary Material



## Data Availability

Additional research data supporting this publication are available as Supporting Information at DOI: 10.25377/sussex.28870451

## Notes

Synthesis; spectroscopic characterization; crystallography details; magnetic measurements; computational details (PDF)
