# Supplementary material for: A Linear Dysprosium(II) Metallocene with a High Effective Energy Barrier and Magnetic Hysteresis up to 70 Kelvin
Source: J Am Chem Soc. 2025 May 15;147(21):18307–16. doi: 10.1021/jacs.5c06222 (PMC12123614; doi:10.1021/jacs.5c06222)
Supplement: Supplementary file 1 [file ja5c06222_si_001.pdf]

Supplementary Information for

A Linear Dysprosium(II) Metallocene with a High Effective Energy Barrier and Magnetic Hysteresis up to 70 Kelvin

Ming Liu,[a] Yan-Cong Chen,[b] Huan Wang,[a] Tao Shang,[a]
Ming-Liang Tong,*[b] Richard A. Layfield,*[c] Akseli Mansikkamäki,*[d] and Fu-Sheng Guo*[a]

a. Institute of Fundamental and Frontier Sciences, University of Electronic Science and Technology of China, Xiyuan Avenue 2006, Chengdu 611731, China.
E-mail: guofush@hotmail.com

b. Key Laboratory of Bioinorganic and Synthetic Chemistry of the Ministry of Education, School of Chemistry, IGCME, GBRCE for Functional Molecular Engineering, Sun Yat-Sen University, Guangzhou 510006, China.
E-mail: tongml@mail.sysu.edu.cn

c. Department of Chemistry, School of Life Sciences, University of Sussex, Brighton, BN1 9QR, U.K.
E-mail: R.Layfield@sussex.ac.uk

d. NMR Research Unit, University of Oulu, P. O. Box 8000, FI-90014, Finland.
E-mail: akseli.mansikkamaki@oulu.fi

Contents

1. General Considerations and Synthesis Procedures S2
2. FTIR spectroscopy S3
3. X-ray crystallography S4-S5
4. UV/vis/NIR Spectroscopy S6-S10
5. Magnetic Measurements S11-S22
6. Computational Details S22-S31
7. References S32-S33

General Considerations

All experiments were conducted under rigorous anhydrous, anaerobic conditions under an atmosphere of purified argon (99.9995%) using standard Schlenk line or glovebox techniques. Solvents were purified by a MIKROUNA solvent purification system and dried over activated 4 Å molecular sieves before use. $[(C_5{}^iPr_5)Dy(Cp^*)BH_4]$ and KC_8 were prepared according to literature procedures.[1,2]

Elemental analyses were performed on a Vario Micro Cube automatic element analyzer. FTIR spectra were recorded on a Bruker Alpha II FTIR spectrometer equipped with a Platinum ATR module. UV/vis/NIR absorption spectra were collected from 200-1000 nm on a Shimadzu UV-3600 Plus spectrophotometer at room temperature.

Synthesis of $[(\eta^5\text{-}C_5{}^iPr_5)Dy(\eta^5\text{-}Cp^*)]\cdot 0.5$hexane

Benzene (30 ml) was added to an ampoule containing $[(\eta^5\text{-}C_5{}^iPr_5)Dy(\eta^5\text{-}Cp^*)(BH_4)]$ (590 mg, 1.0 mmol), KC_8 (201 mg , 1.5 mmol) and a glass coated stirrer bar. The reaction mixture was stirred for three days at room temperature, resulting in a red solution and a black precipitate. After the solvent was removed under vacuum, the residue was extracted into *n*-hexane (3 × 15 mL) and filtered. After removing the solvent, a dark red powder was obtained. Recrystallization from *n*-hexane at –35°C overnight gave red crystals (yield: 245 mg, 40%). The compound does not show any signs of decomposition in the solid-state following storage under argon atmosphere at room temperature for several months. ***Elemental analysis*** [%] Calculated for $[(\eta^5\text{-}C_5{}^iPr_5)Dy(\eta^5\text{-}Cp^*)]\cdot 0.3$hexane: C 63.76 %, H 9.12 %. Found: C 63.77 %, H 9.06 %. ***FTIR*** (cm^{-1}): 2972s, 2962s, 2929s, 2866s, 2720w, 1866w, 1632w, 1548w, 1453s, 1439s, 1375s, 1361s, 1310m, 1250w, 1158s, 1111m, 1082s, 1020w, 905w, 799w, 762w, 741w, 702w, 663w, 587w, 546w, 517m, 487s, 448w, 425w.

FTIR Spectroscopy

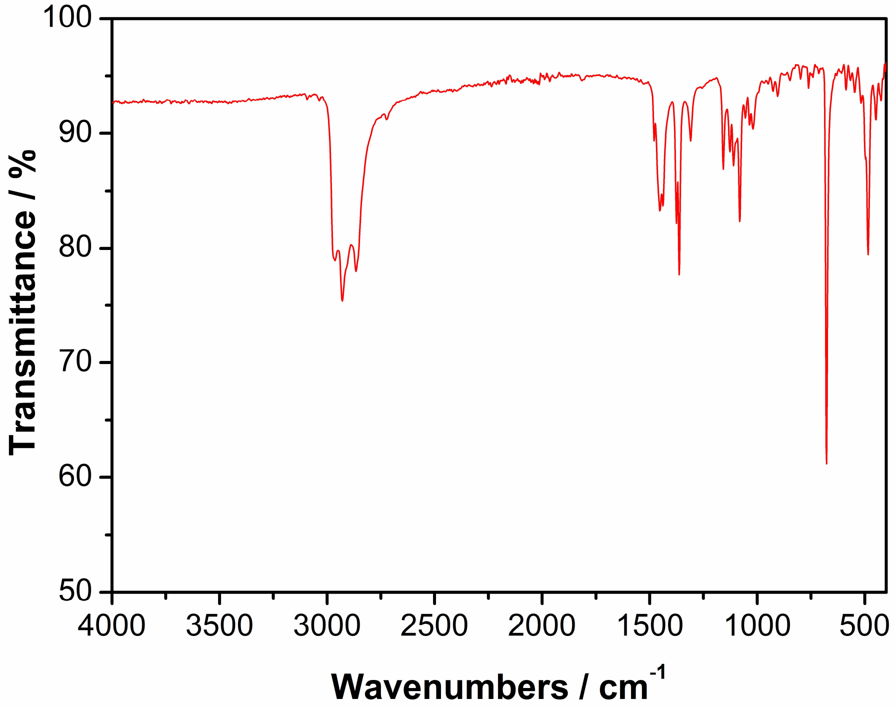

Figure S1. FTIR spectrum of [(η^5-$C_5{}^iPr_5$)Dy(η^5-Cp*)]·0.5hexane.

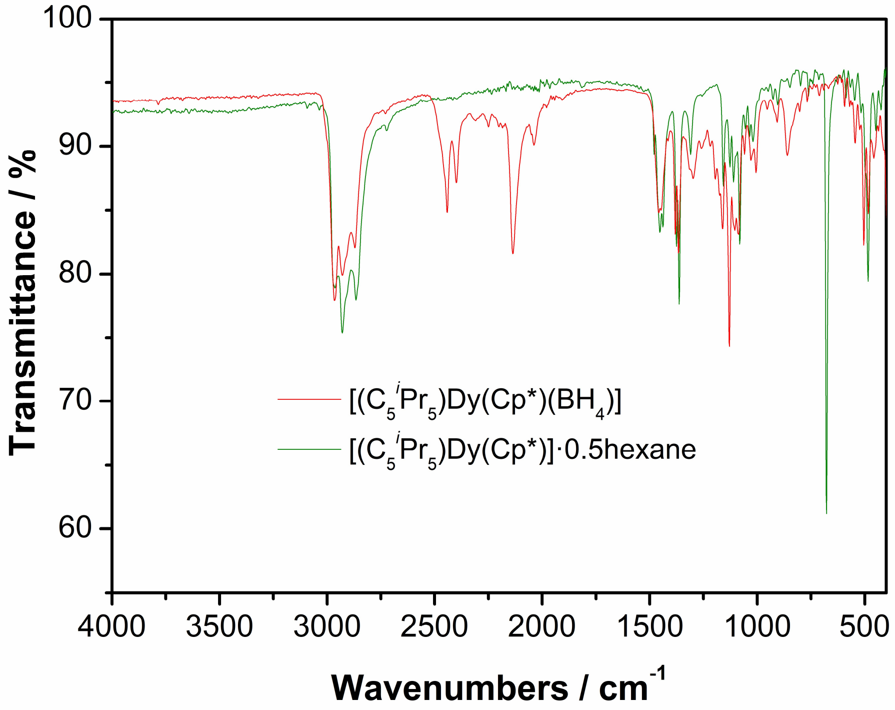

Figure S2. Comparison of FTIR spectrum of [(η^5-$C_5{}^iPr_5$)Dy(η^5-Cp*)]·0.5hexane and [(η^5-$C_5{}^iPr_5$)Dy(η^5-Cp*)(BH_4)].[1]

X-ray crystallography

X-ray diffraction data were collected on Bruker APEX-II CCD diffractometer. Data collection and processing (cell refinement, data reduction and absorption) were performed using the program *APEX 3*. Structures were solved in Olex2 with SHELXT using intrinsic phasing and were refined with SHELXL using least squares minimisation.[3-5] Anisotropic thermal parameters were used for the non-hydrogen atoms and isotropic parameters for the hydrogen atoms. Hydrogen atoms were added geometrically and refined using a riding model.

The $C_5{}^iPr_5$ ligand in the structure is disordered and was refined assuming all the isopropyl groups disorder over two opposite orientations, yielding a ratio of 0.549:0.451 for the two components. The distances (C9-C2, C9A-C2, C3-C12, C3-C12A, C4-C15, C4-C15A, C5-C18, C5-C18A, C6-C1, C6A-C1) between the ^{i}Pr groups and the Cp ring were fixed to be equal using a SADI restraint. The five methyl carbons (C26, C27, C28, C29 and C30) in the Cp* ring could not be stabilized unless anisotropic displacement parameters were forced to behave in an isotropic manner. The lattice hexane is disordered and was refined assuming C32 and C33 disorder over two opposite orientations, yielding a ratio of 0.374:0.626 for the two components.

Table S1. Crystal data and structure refinement for $[(\eta^5\text{-}C_5{}^iPr_5)Dy(\eta^5\text{-}Cp^*)]\cdot 0.5$hexane.

	$[(\eta^5\text{-}C_5{}^iPr_5)Dy(\eta^5\text{-}Cp^*)]\cdot 0.5$hexane
CCDC	2443266
Empirical formula	$C_{33}H_{57}Dy$
Formula weight	616.28
Crystal system	triclinic
Space group	*P*-1
a/Å	10.0007(11)
b/Å	10.0166(13)
c/Å	18.229(2)
α/°	75.566(4)
β/°	75.058(4)
γ/°	71.109(4)
V/Å^3	1641.6(4)
Temperature/K	150
Z	2
ρ_{calc}/g cm^{-3}	1.247
Crystal size/mm^3	0.47 × 0.2 × 0.14
Radiation	Mo-K$_\alpha$ (λ = 0.71073)
2θ range/°	4.37 to 51.992
Reflections collected	38167
Independent reflections	6444 [R_{int} = 0.0620, R_{sigma} = 0.0360]
Data/restraints/parameters	6444/105/387
Goodness-of-fit on F^2	1.179
Final *R* indexes [*I* >=2σ (I)]	R_1 = 0.0345, wR_2 = 0.0784
Final *R* indexes [all data]	R_1 = 0.0397, wR_2 = 0.0801

Table S2. Selected bond lengths (Å) and angle (°) in [(η^5-$C_5{}^iPr_5$)Dy(η^5-Cp*)]·0.5hexane.

Dy1-C1	2.644(4) Å
Dy1-C2	2.638(4) Å
Dy1-C3	2.640(4) Å
Dy1-C4	2.648(4) Å
Dy1-C5	2.657(4) Å
Dy1–Cp^{iPr5}_{cent}	2.3587(3) Å
Dy1-C21	2.622(4) Å
Dy1-C22	2.612(5) Å
Dy1-C23	2.608(5) Å
Dy1-C24	2.619(4) Å
Dy1-C25	2.639(4) Å
Dy1–Cp^*_{cent}	2.3376(3) Å
Cp^{iPr5}_{cent}-Dy-Cp^*_{cent}	179.643(9)°

UV/vis/NIR Spectroscopy

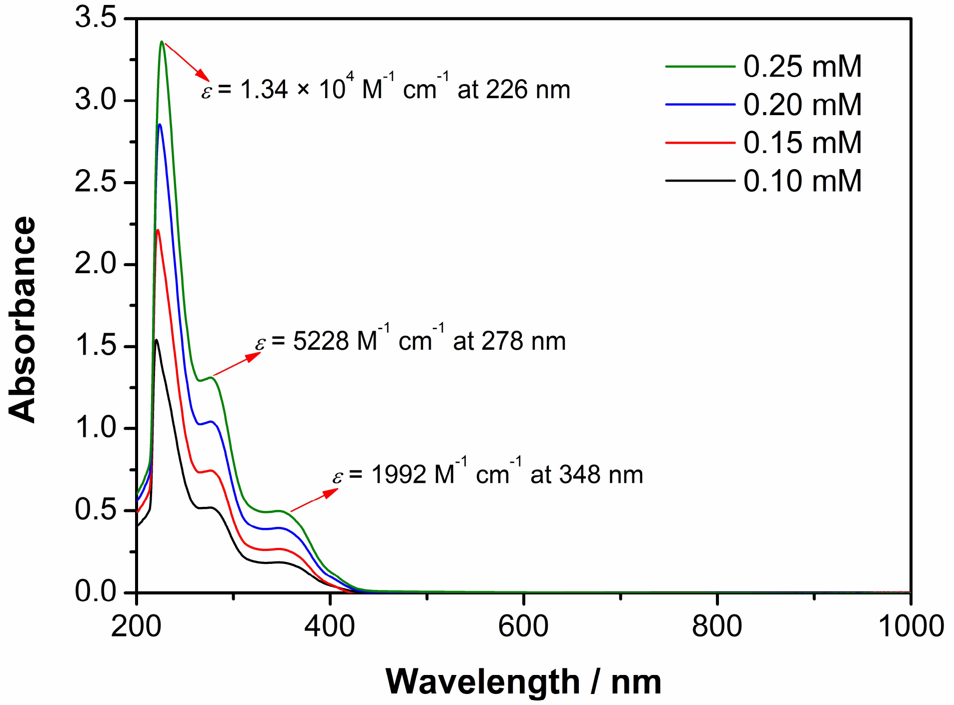

Figure S3. UV/vis/NIR spectrum of [(η^5-$C_5{}^iPr_5$)Dy(η^5-Cp*)(BH_4)] in hexane at varying concentrations.

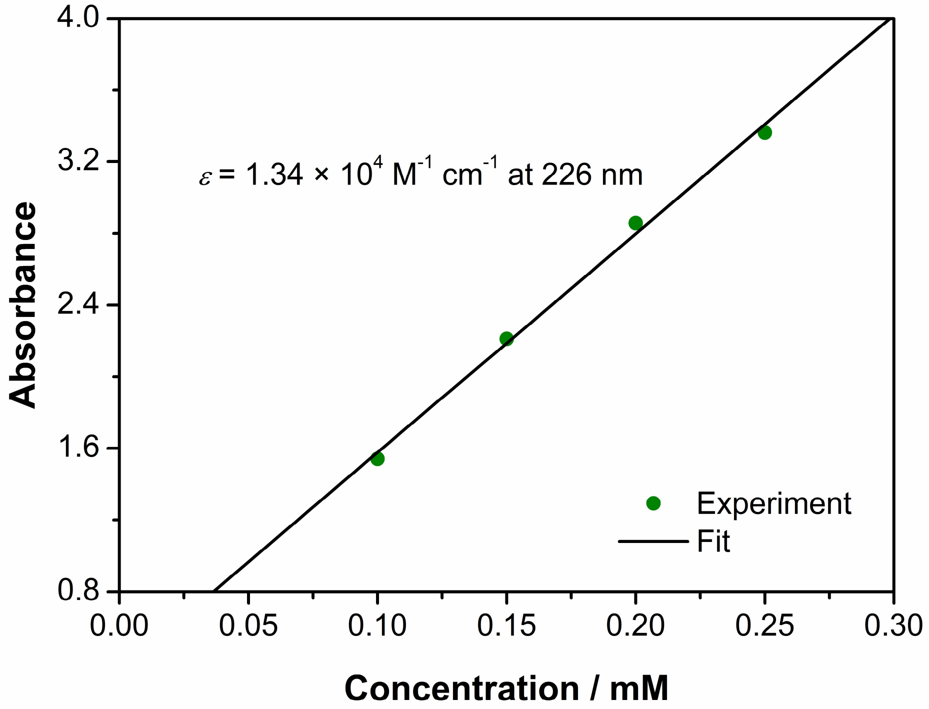

Figure S4. Plot of absorbance versus concentration for [(η^5-$C_5{}^iPr_5$)Dy(η^5-Cp*)(BH_4)] at 226 nm in the UV/vis/NIR spectrum. The green points are from the UV/vis/NIR spectrum (Figure S3). The solid black line is the best fit to the data to extract the extinction coefficient.

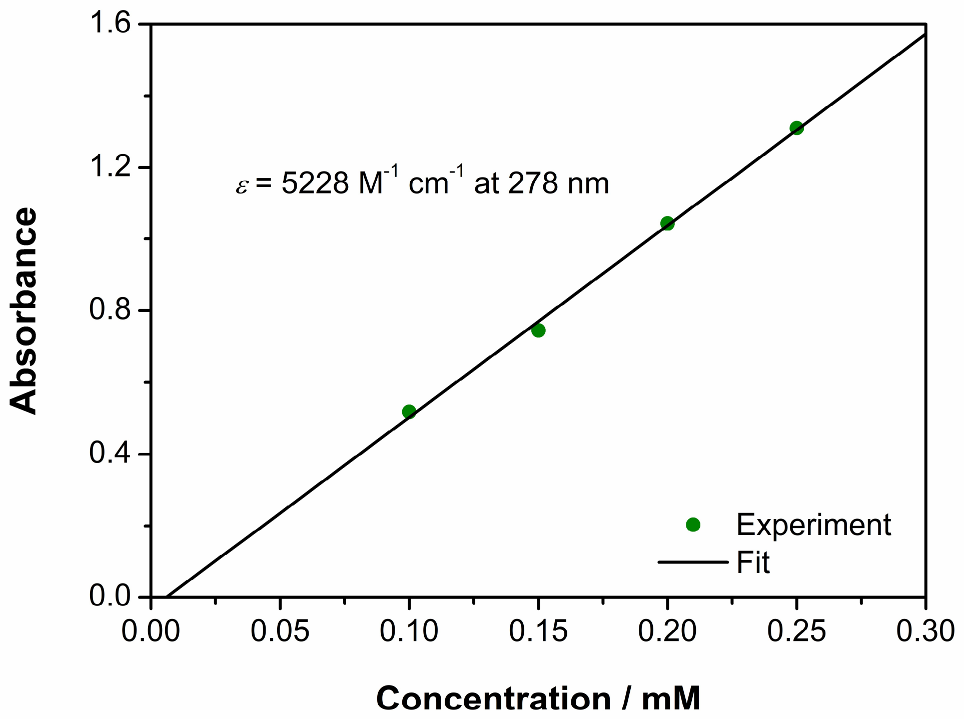

Figure S5. Plot of absorbance versus concentration for [(η^5-$C_5{}^iPr_5$)Dy(η^5-Cp*)(BH_4)] at 278 nm in the UV/vis/NIR spectrum. The green points are from the UV/vis/NIR spectrum (Figure S3). The solid black line is the best fit to the data to extract the extinction coefficient.

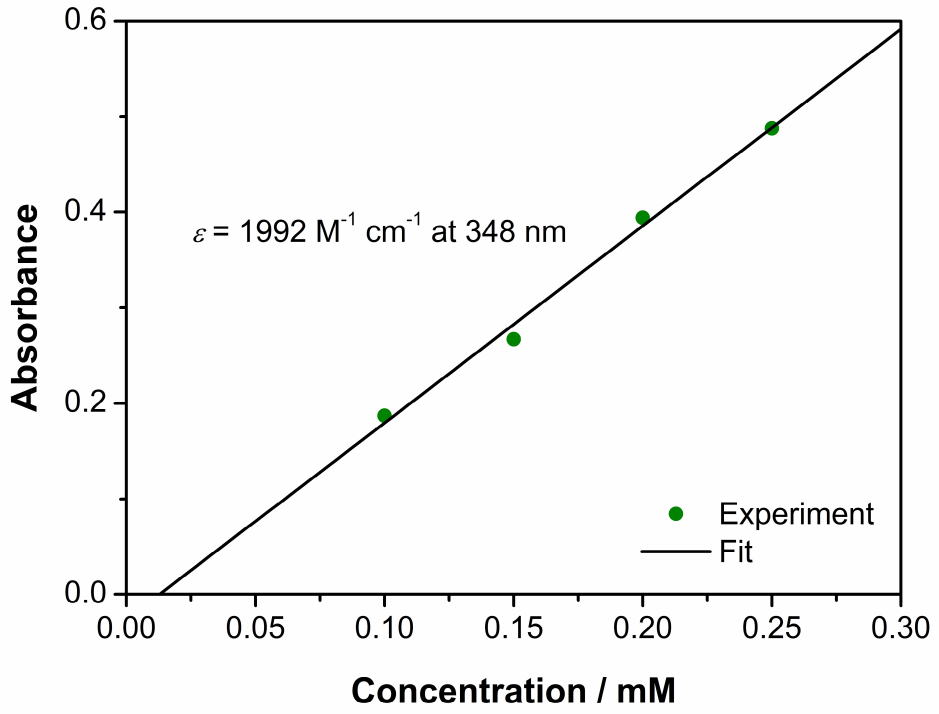

Figure S6. Plot of absorbance versus concentration for [(η^5-$C_5{}^iPr_5$)Dy(η^5-Cp*)(BH_4)] at 348 nm in the UV/vis/NIR spectrum. The green points are from the UV/vis/NIR spectrum (Figure S3). The solid black line is the best fit to the data to extract the extinction coefficient.

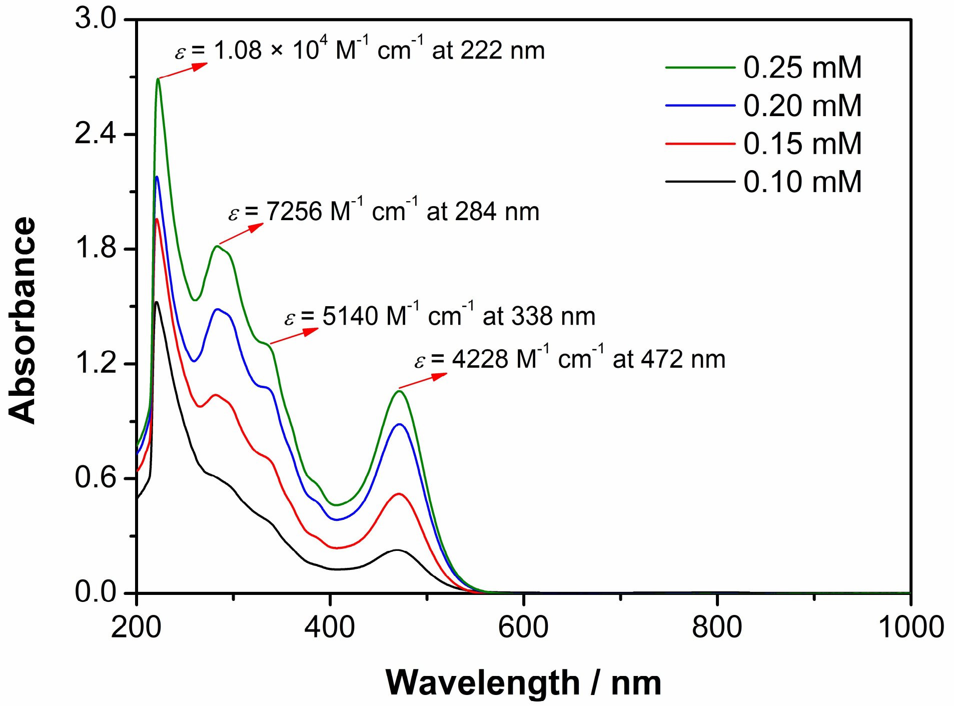

Figure S7. UV/vis/NIR spectrum of [(η^5-$C_5{}^iPr_5$)Dy(η^5-Cp*)]·0.5hexane in hexane at varying concentrations.

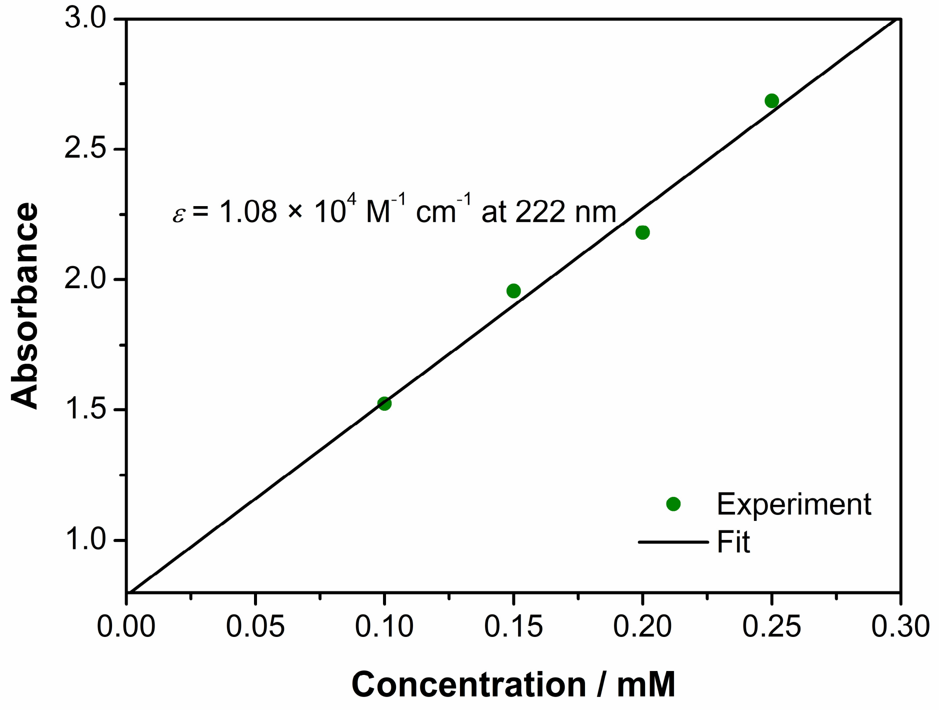

Figure S8. Plot of absorbance versus concentration for [(η^5-$C_5{}^iPr_5$)Dy(η^5-Cp*)]·0.5hexane at 222 nm in the UV/vis/NIR spectrum. The green points are from the UV/vis/NIR spectrum (Figure S7). The solid black line is the best fit to the data to extract the extinction coefficient.

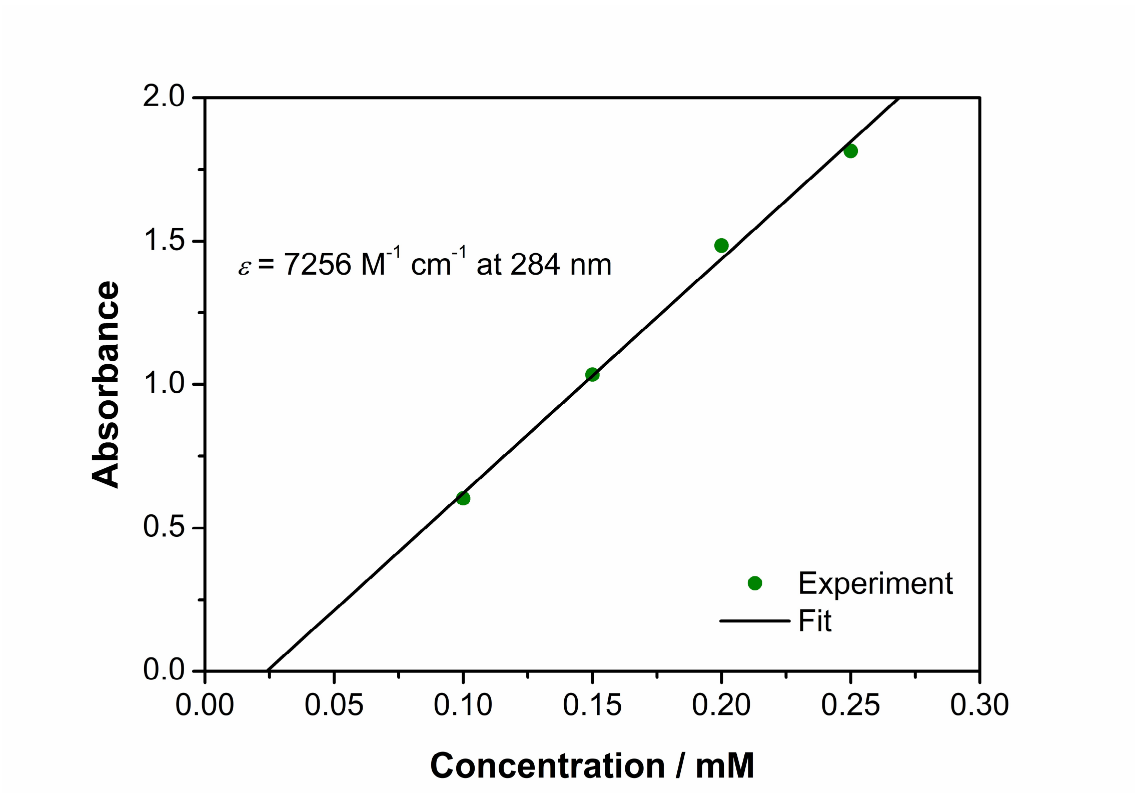

Figure S9. Plot of absorbance versus concentration for [(η^5-$C_5{}^iPr_5$)Dy(η^5-Cp*)]·0.5hexane at 284 nm in the UV/vis/NIR spectrum. The green points are from the UV/vis/NIR spectrum (Figure S7). The solid black line is the best fit to the data to extract the extinction coefficient.

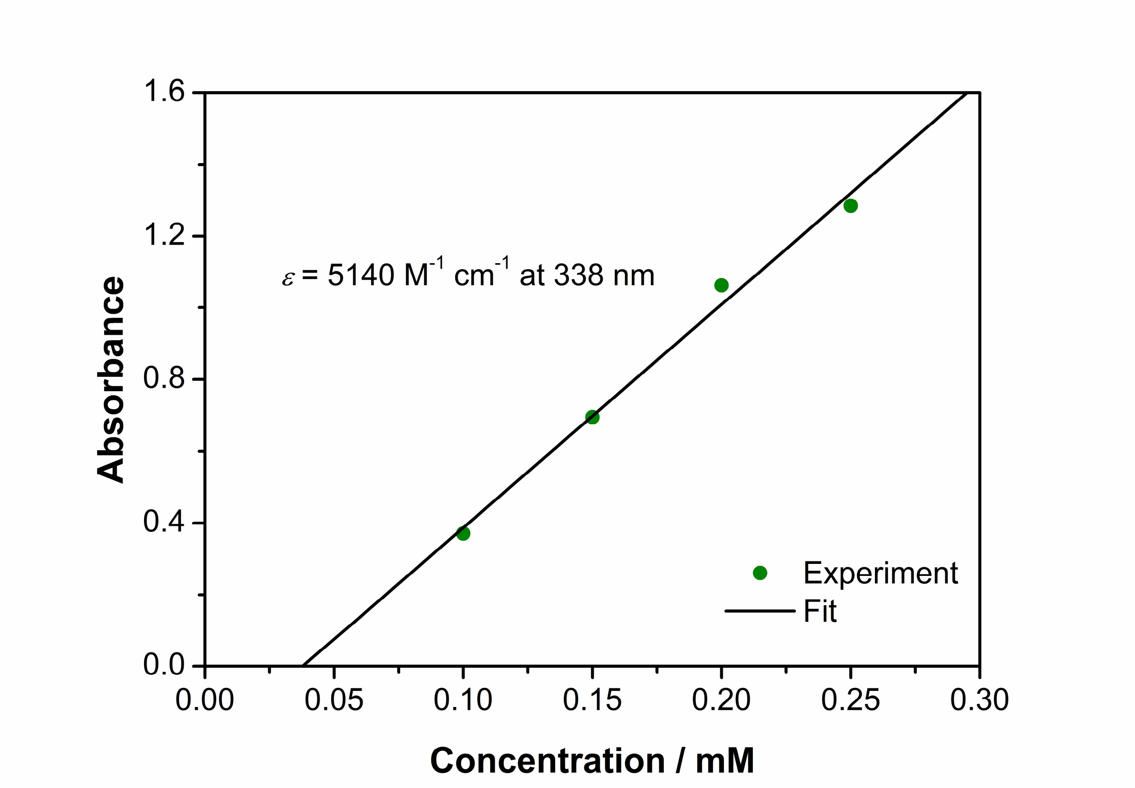

Figure S10. Plot of absorbance versus concentration for [(η^5-$C_5{}^iPr_5$)Dy(η^5-Cp*)]·0.5hexane at 338 nm in the UV/vis/NIR spectrum. The green points are from the UV/vis/NIR spectrum (Figure S7). The solid black line is the best fit to the data to extract the extinction coefficient.

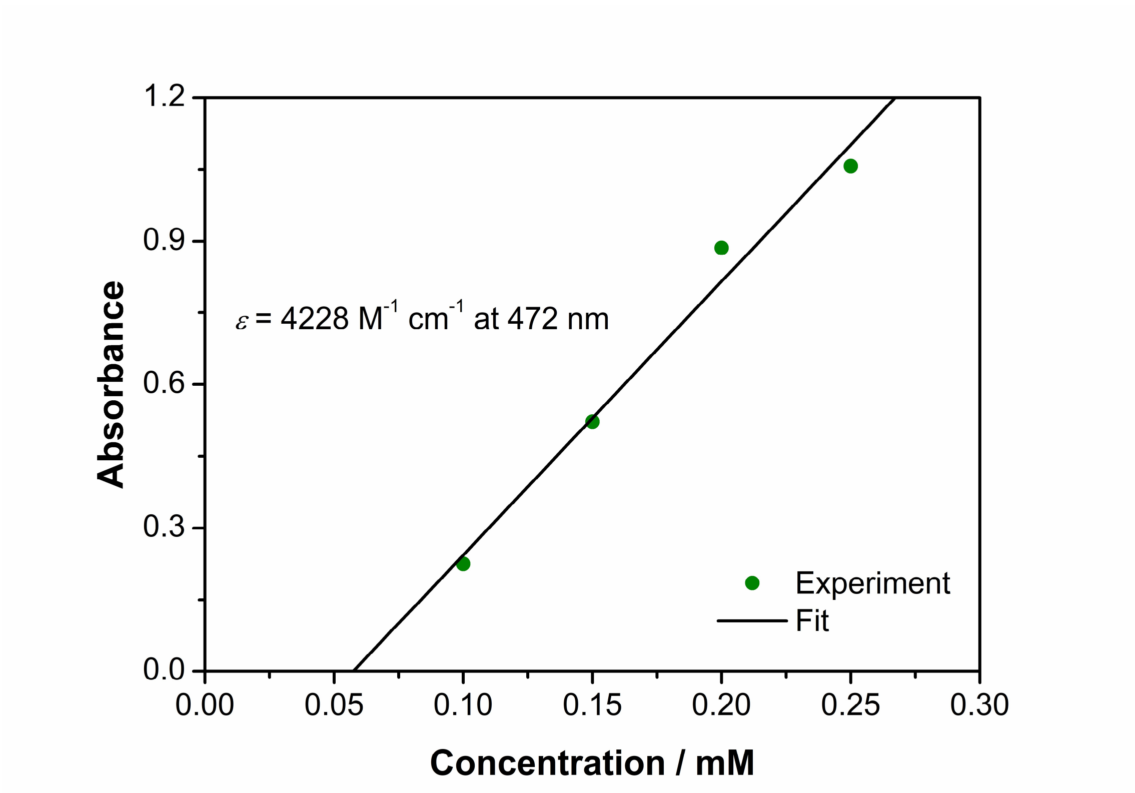

Figure S11. Plot of absorbance versus concentration for [(η^5-$C_5{}^iPr_5$)Dy(η^5-Cp*)]·0.5hexane at 338 nm in the UV/vis/NIR spectrum. The green points are from the UV/vis/NIR spectrum (Figure S7). The solid black line is the best fit to the data to extract the extinction coefficient.

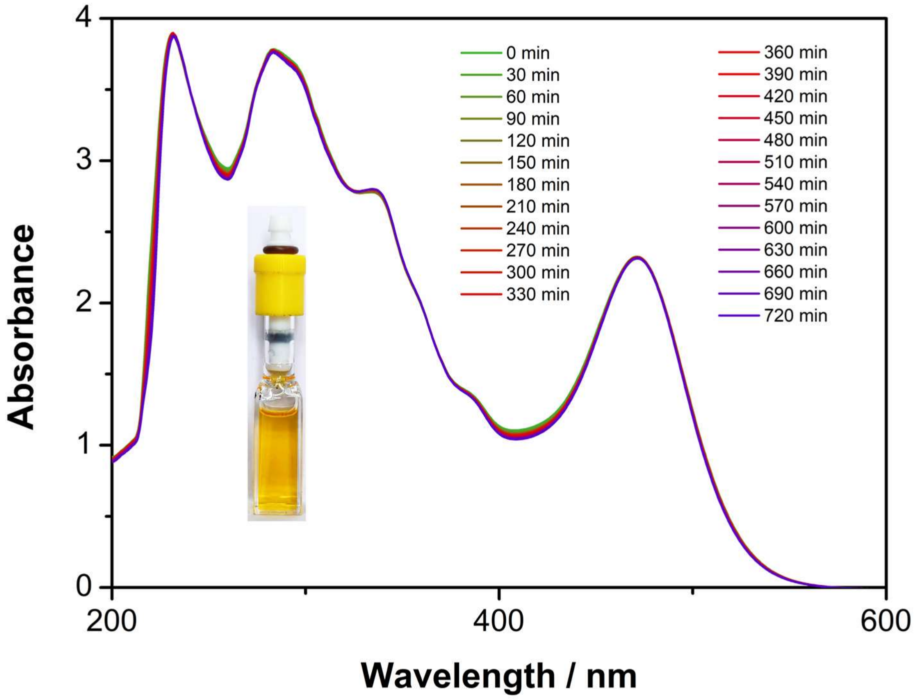

Figure S12. UV/vis/NIR spectra of [(η^5-$C_5{}^iPr_5$)Dy(η^5-Cp*)]·0.5hexane in hexane at room temperature collected over 720 minutes. The inset shows the hexane solution of [(η^5-$C_5{}^iPr_5$)Dy(η^5-Cp*)]·0.5hexane.

Magnetic Measurements

In an argon-filled glovebox, [(η^5-$C_5{}^iPr_5$)Dy(η^5-Cp*)]·0.5hexane (29.9 mg) was loaded into an NMR tube (OD: 5 mm; ID: 4mm). Fomblin® Y (LVAC 25/6) (77.4 mg) was then added carefully by a syringe with a long needle. Drinking straws were used during sample addition to mitigate the effects of static. The tube was fitted with an O-ring sealable adapter, transferred from the glovebox, and connected to a vacuum pump. After cooling the bottom half of the tube to –78°C, the tube was sealed to a length of about 6 cm. An empty tube of similar length was placed below the sample tube in the sample holder. Prior to centring and measurement, the sample was first cooled in zero field below the pour point of the Fomblin oil to prevent crystal orientation. Direct current (DC) magnetic susceptibility and magnetization data (VSM mode) were then collected using a Quantum Design MPMS3 magnetometer in warming mode. Alternating current (AC) magnetic susceptibility measurements were performed using a Quantum Design MPMS3 magnetometer using an oscillating field of 2 Oe. Diamagnetic corrections were performed on the sample and the Fomblin using Pascal's constants.[6]

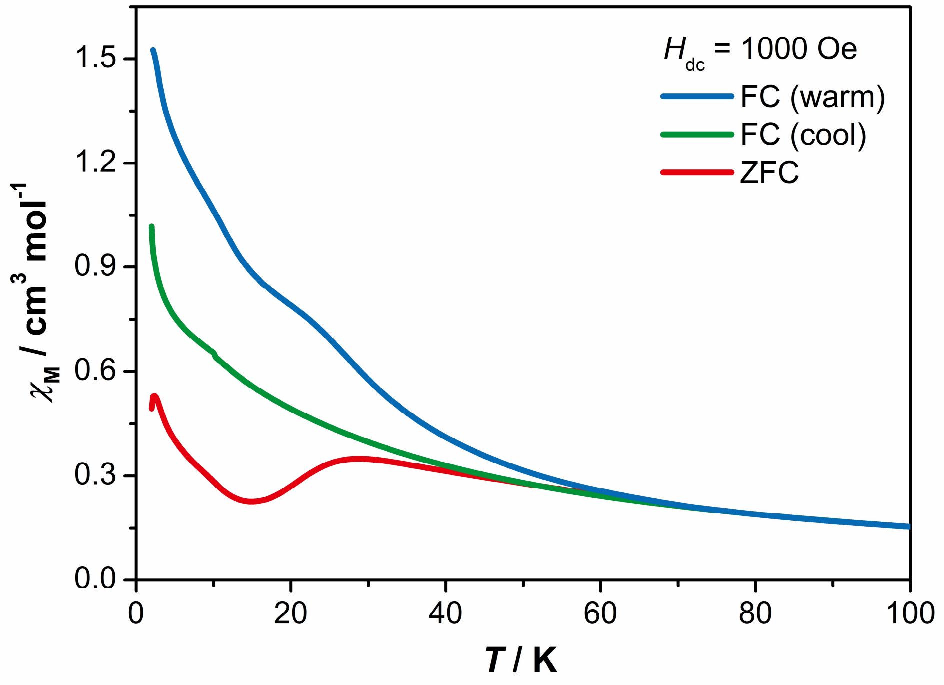

Figure S13. Field-cooled (FC, blue line) and zero-field-cooled (ZFC, red line) variable-temperature magnetic susceptibility for [(η^5-$C_5{}^iPr_5$)Dy(η^5-Cp*)]·0.5hexane under 1000 Oe DC filed in warming mode (2 K min^{-1}) from 2 to 100 K. The green line represents the FC variable-temperature magnetic susceptibility in cool mode (2 K min^{-1}) from 100 to 2 K.

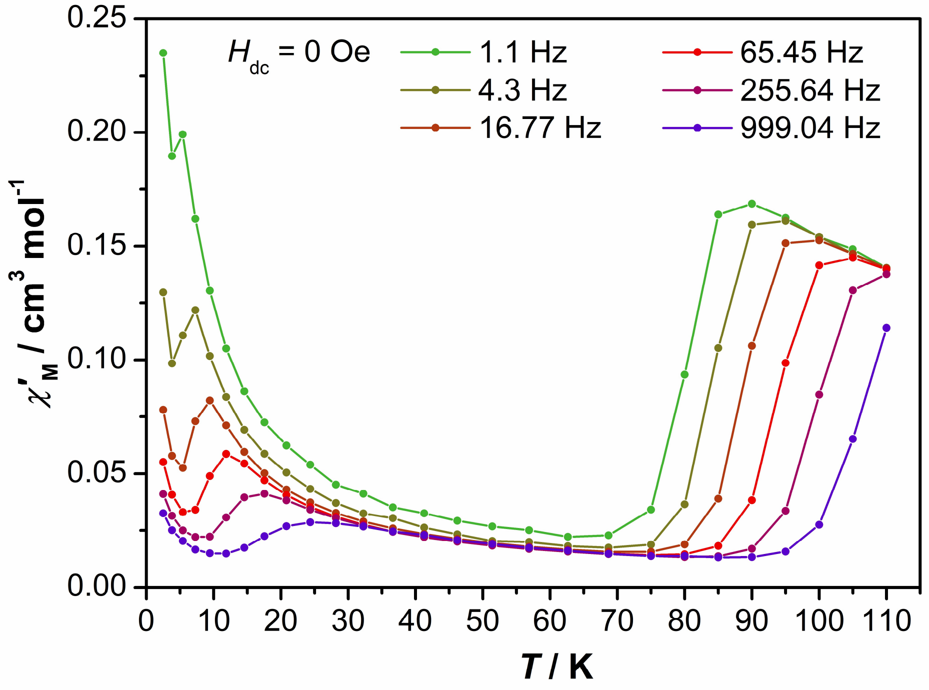

Figure S14. Temperature dependence of the in-phase susceptibility χ'_M for $[(\eta^5\text{-}C_5{}^i Pr_5)Dy(\eta^5\text{-}Cp^*)]\cdot 0.5$hexane, collected under zero DC field at AC frequencies of ν = 1.1-999 Hz from 2-110 K. Solid lines are a guide to the eye.

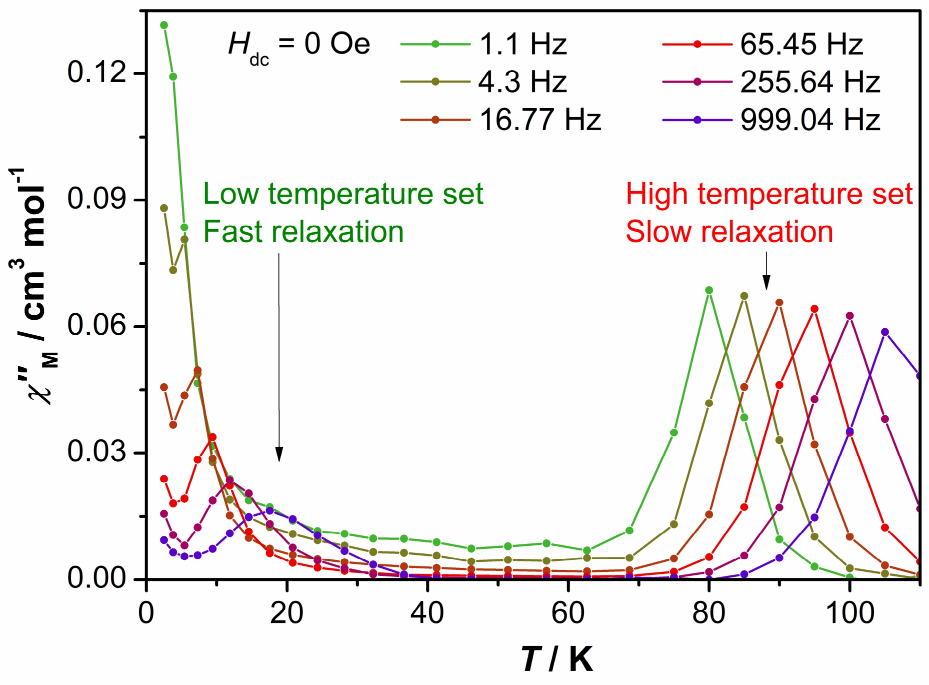

Figure S15. Temperature dependence of the out-of-phase susceptibility χ''_M for $[(\eta^5\text{-}C_5{}^i Pr_5)Dy(\eta^5\text{-}Cp^*)]\cdot 0.5$hexane, collected under zero DC field at AC frequencies of ν = 1.1-999 Hz from 2-110 K. Solid lines are a guide to the eye.

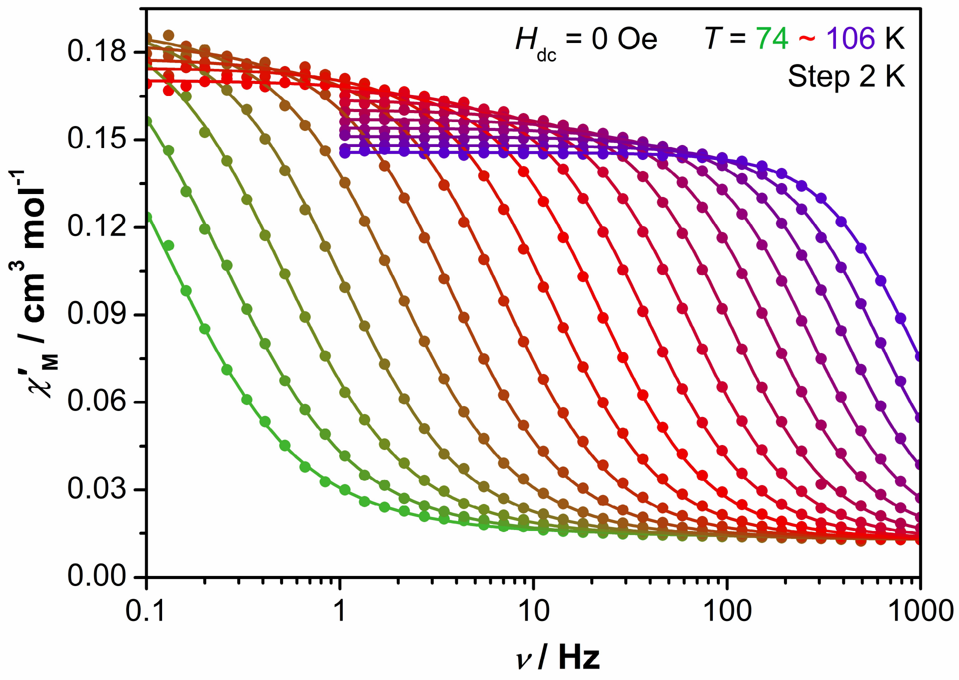

Figure S16. Frequency dependence of the in-phase susceptibility (χ'_M) for [(η^5-$C_5{}^iPr_5$)Dy(η^5-Cp*)]·0.5hexane in zero DC field at AC frequencies of 0.1-999 Hz from 74-106 K. Solid lines represent fits to the data using a generalized Debye equation.

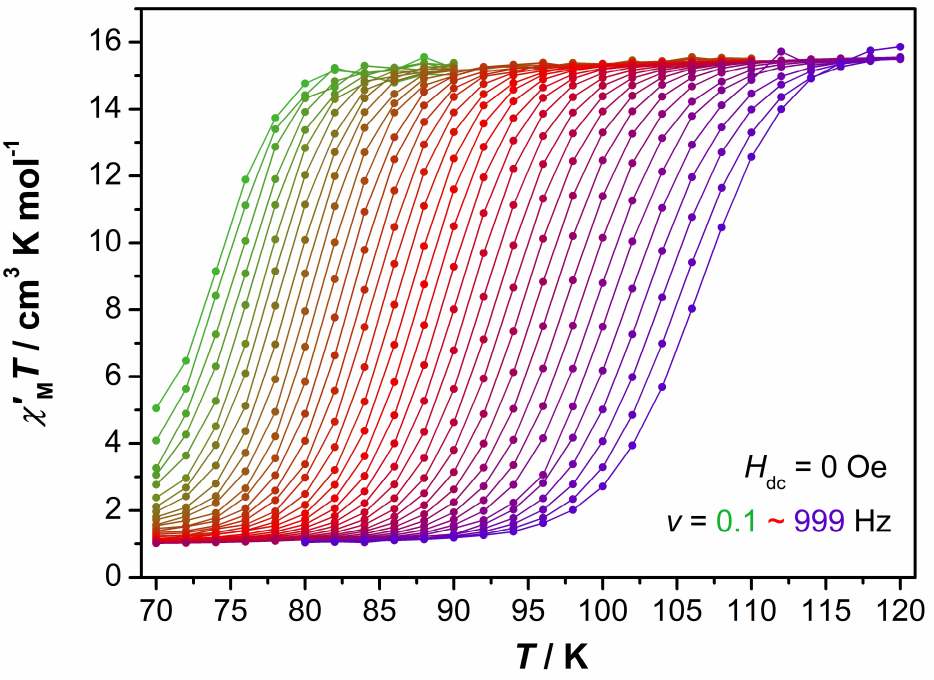

Figure S17. Temperature dependence of the in-phase $\chi'_M T$ product for [(η^5-$C_5{}^iPr_5$)Dy(η^5-Cp*)]·0.5hexane, collected under zero DC field at AC frequencies of ν = 0.1-999 Hz from 70-120 K. Solid lines are a guide to the eye.

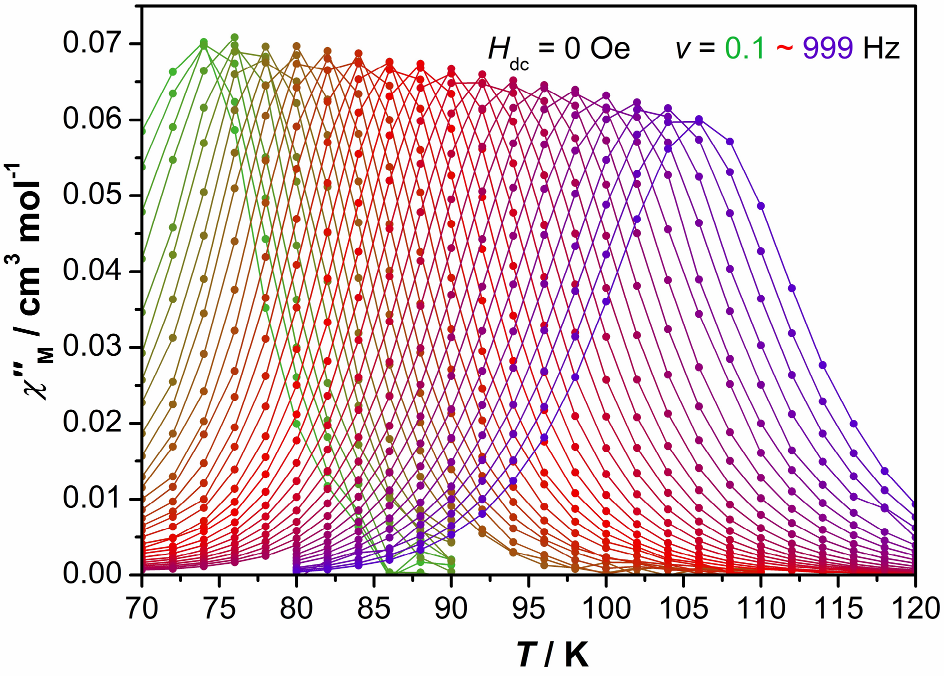

Figure S18. Temperature dependence of the out-of-phase susceptibility χ''_M for [(η^5-$C_5{}^iPr_5$)Dy(η^5-Cp*)]·0.5hexane, collected under zero DC field at AC frequencies of ν = 0.1-999 Hz from 70-120 K. Solid lines are a guide to the eye.

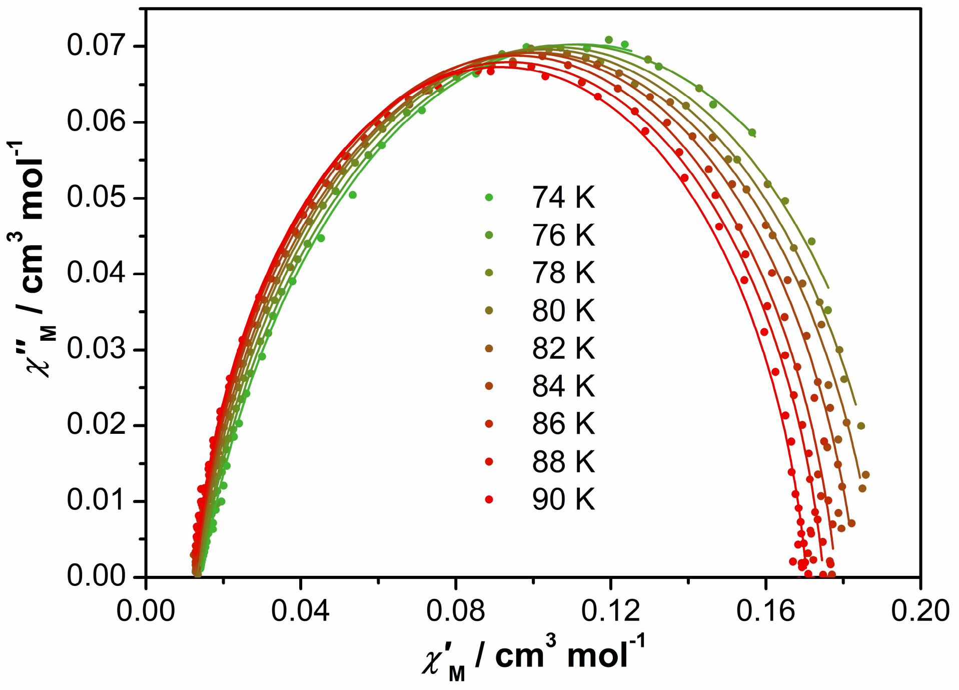

Figure S19. Cole-Cole plots for the AC susceptibilities in zero DC field for [(η^5-$C_5{}^iPr_5$)Dy(η^5-Cp*)]·0.5hexane from 74-90 K. Solid lines represent fits to the data using a generalized Debye equation.

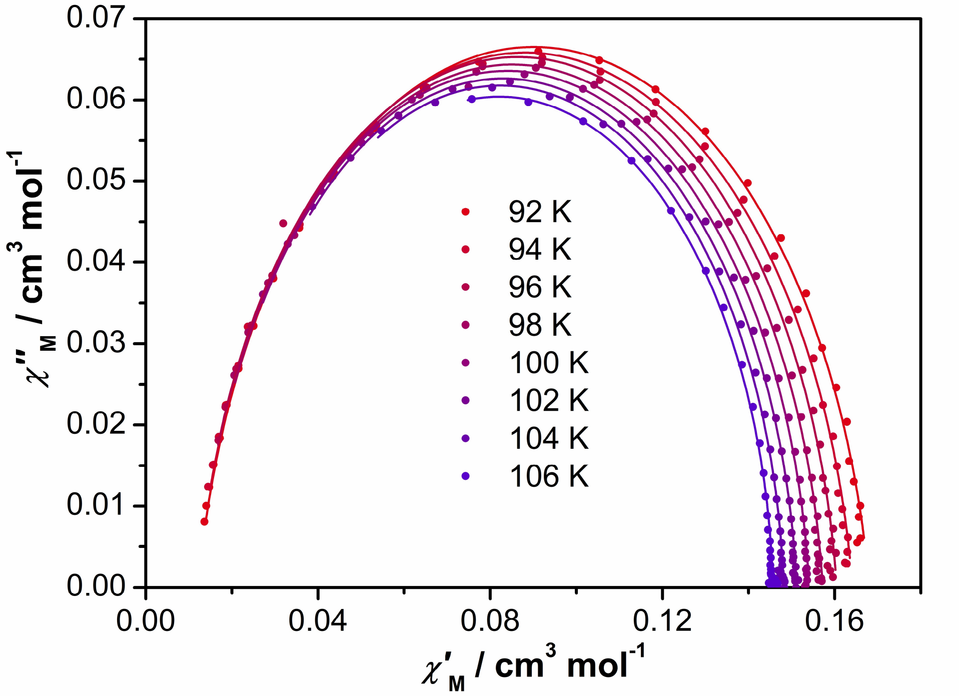

Figure S20. Cole-Cole plots for the AC susceptibilities in zero DC field for [(η^5-$C_5{}^iPr_5$)Dy(η^5-Cp*)]·0.5hexane from 92-106 K. Solid lines represent fits to the data using a generalized Debye equation.

Table S3. Relaxation fitting parameters for [(η^5-C$_5$^{i}Pr$_5$)Dy(η^5-Cp*)]·0.5hexane corresponding to Figures S19 and S20 using the generalized Debye model.

T / K	χ_∞ / cm^3 mol^{-1}	χ_S / cm^3 mol^{-1}	α	τ / s
74	0.20892(0.00216)	0.01435(2.165E-4)	0.20317(0.00501)	1.25996(0.02607)
76	0.20504(9.06663E-4)	0.01373(1.75917E-4)	0.19352(0.00303)	0.65151(0.0058)
78	0.19686(7.22702E-4)	0.0137(2.55025E-4)	0.16987(0.0035)	0.31429(0.00243)
80	0.19239(4.2451E-4)	0.01315(0.01315)	0.15931(0.0025)	0.15709(8.36823E-4)
82	0.1886(3.69038E-4)	0.01279(1.99765E-4)	0.15095(0.00264)	0.08171(4.39353E-4)
84	0.18334(3.11982E-4)	0.01276(2.21035E-4)	0.13228(0.00269)	0.04231(2.22393E-4)
86	0.17812(4.84722E-4)	0.01291(4.29796E-4)	0.11571(0.00488)	0.02271(2.10978E-4)
88	0.17493(3.10894E-4)	0.01252(3.35667E-4)	0.11255(0.00352)	0.01269(8.4616E-5)
90	0.17049(2.47065E-4)	0.01252(3.23164E-4)	0.10104(0.00317)	0.00718(4.2262E-5)
92	0.16801(2.40169E-4)	0.01221(2.58301E-4)	0.10022(0.00251)	0.00421(1.84998E-5)
94	0.16412(2.16736E-4)	0.01221(2.99561E-4)	0.09089(0.00263)	0.00251(1.14536E-5)
96	0.16048(2.19799E-4)	0.01236(3.91051E-4)	0.08016(0.0031)	0.00153(8.15474E-6)
98	0.15721(1.55836E-4)	0.01307(3.6228E-4)	0.07187(0.00256)	9.52503E-4(4.26858E-6)
100	0.15407(1.44038E-4)	0.01371(4.50604E-4)	0.06261(0.0028)	6.06849E-4(3.1016E-6)
102	0.15115(1.07964E-4)	0.01481(4.73912E-4)	0.05339(0.00254)	3.96374E-4(2.01457E-6)
104	0.14812(1.17812E-4)	0.01653(7.60608E-4)	0.03984(0.00346)	2.65891E-4(2.11742E-6)
106	0.14565(1.00826E-4)	0.01841(0.00104)	0.03282(0.00385)	1.80488E-4(1.98704E-6)

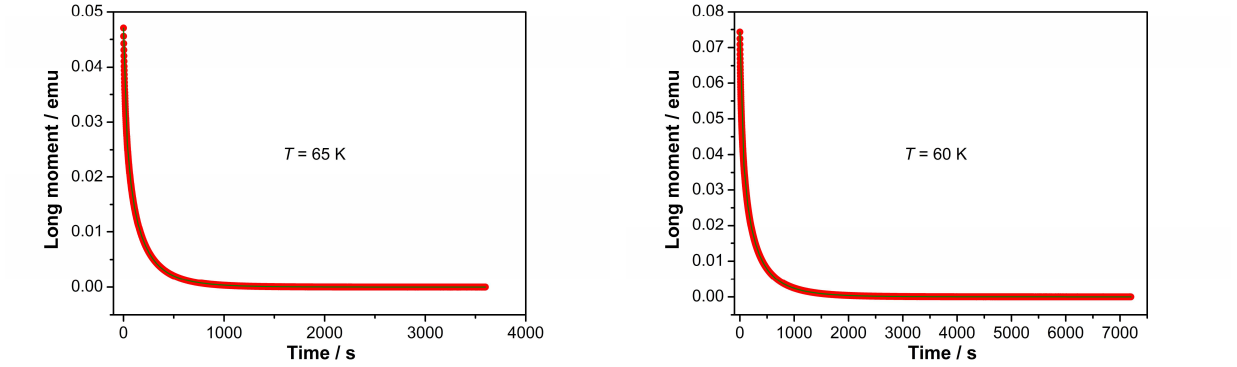

Figure S21. Plot of magnetization decay *vs.* time used to derive DC relaxation times for [(η^5-$C_5{}^iPr_5$)Dy(η^5-Cp*)]·0.5hexane at 65 and 60 K. The solid lines are the best fit to the exponential decay as $M(t) = M_f + (M_0 - M_f)\exp[(-(t/\tau)^{\beta}]$, where M_0 is the initial magnetization, M_f is the final magnetization, τ is the relaxation time, and β is a generalized coefficient, which should be equal to 1 for an ideal exponential decay.

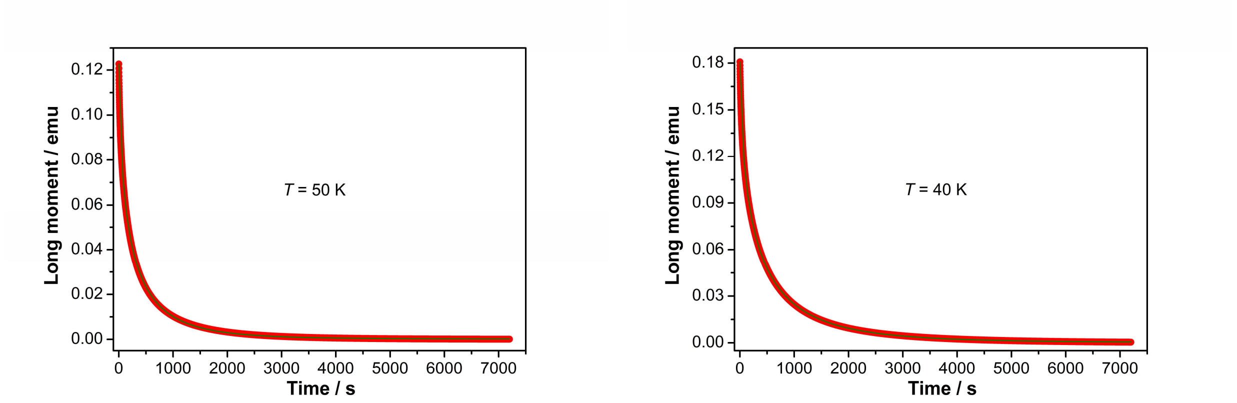

Figure S22. Plot of magnetization decay *vs.* time used to derive relaxation times for [(η^5-$C_5{}^iPr_5$)Dy(η^5-Cp*)]·0.5hexane at 50 and 40 K.

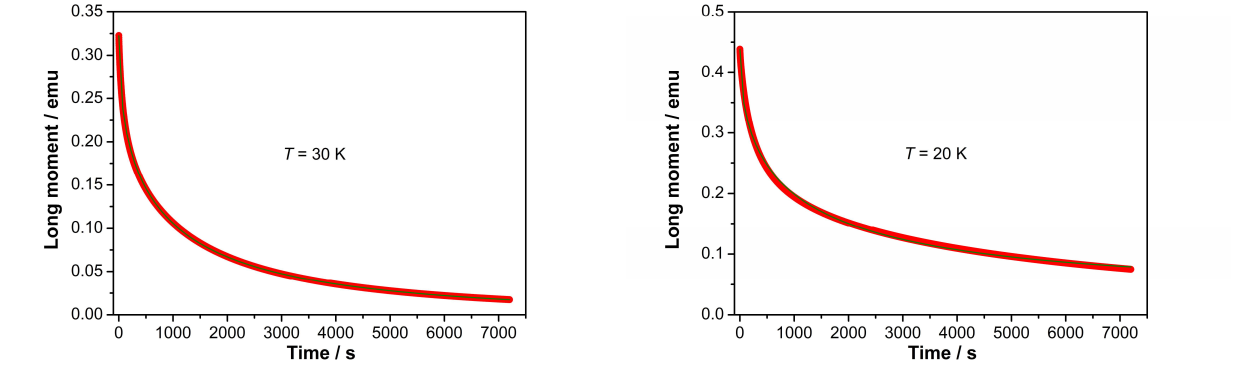

Figure S23. Plot of magnetization decay *vs.* time used to derive relaxation times for [(η^5-$C_5{}^iPr_5$)Dy(η^5-Cp*)]·0.5hexane at 30 and 20 K.

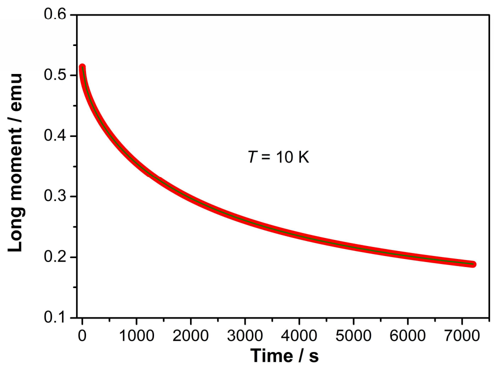

Figure S24. Plot of magnetization decay *vs.* time used to derive relaxation times for [(η^5-$C_5{}^iPr_5$)Dy(η^5-Cp*)]·0.5hexane at 10 K.

Table S4. Fitting parameters (initial magnetization (M_0), final magnetization (M_f), relaxation time (τ), and generalized coefficient (β)) at different temperatures from the least-squares fitting to the exponential decay as $M(t) = M_f + (M_0 - M_f) \exp[(-(t/\tau)^{\beta}]$, as shown in Figures S21-24.

T / K	M_f	M_0	τ / s	β
65	0	0.04704	84.00502	0.63979
60	5.28236E-6	0.07432	131.22877	0.60782
50	2.06784E-4	0.12269	210.35062	0.59136
40	2.87215E-4	0.1808	304.34381	0.58196
30	0.00143	0.32282	785.92239	0.4963
20	0.04707	0.43856	1115.99766	0.49252
10	0.13482	0.51454	2568.19932	0.6455

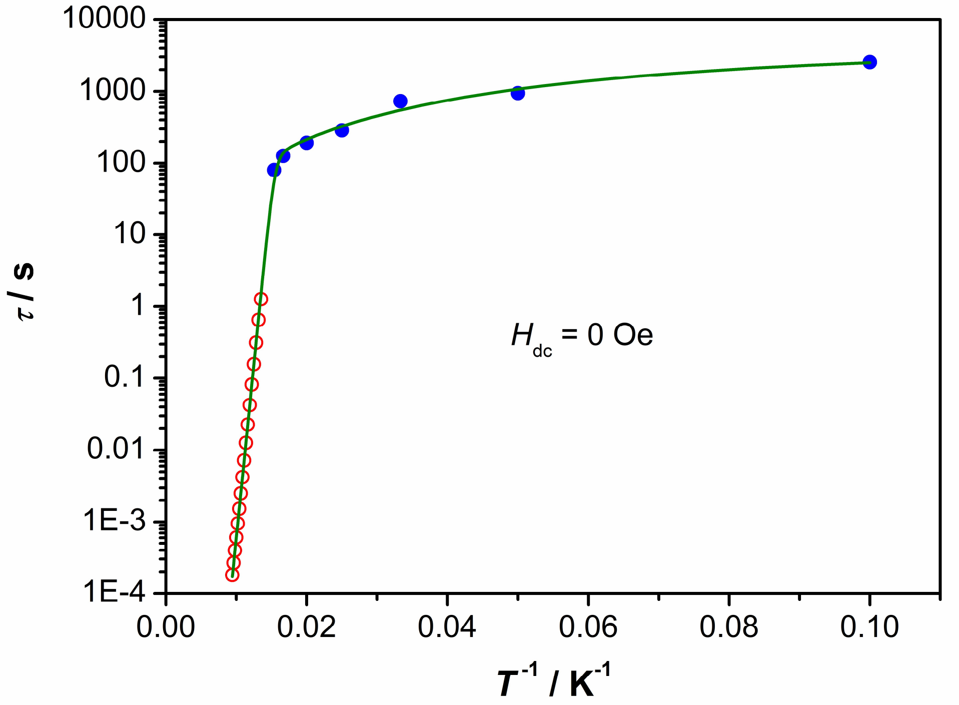

Figure S25. Temperature dependence of the relaxation time for [(η^5-$C_5{}^iPr_5$)Dy(η^5-Cp*)]·0.5hexane. The red points are from the AC susceptibility data, and the blue points are from measurements of the DC magnetic relaxation time. The solid green line is the best fit.

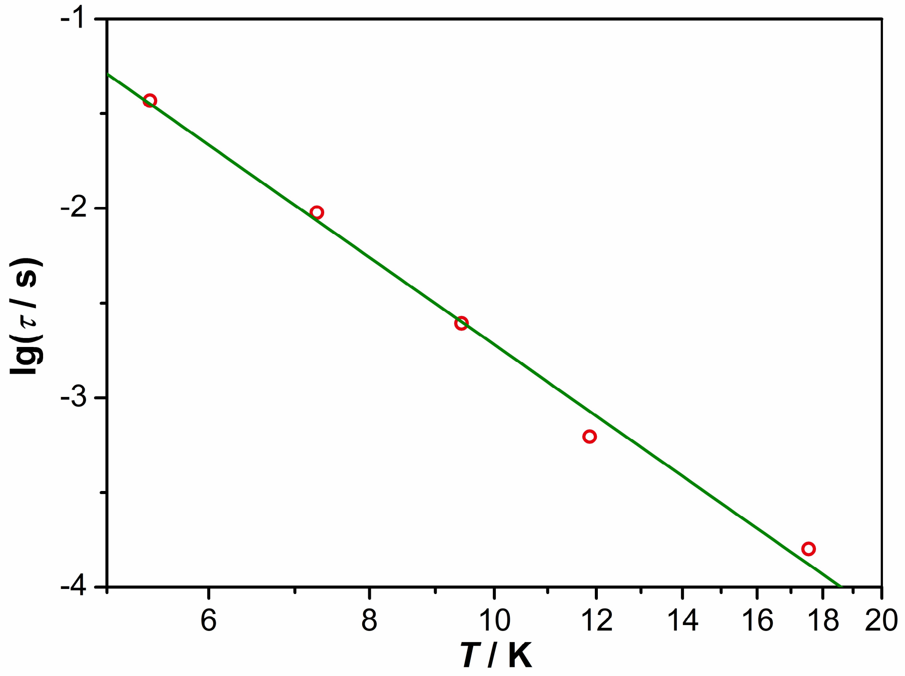

Figure S26. Temperature dependence of the relaxation time as log(τ/s) for [(η^5-$C_5{}^iPr_5$)Dy(η^5-Cp*)]·0.5hexane in the low temperature set. The solid lines are the best fit to the Raman expression $\tau^{-1} = CT^n$ in which C and n are the Raman coefficient and Raman exponent, respectively.

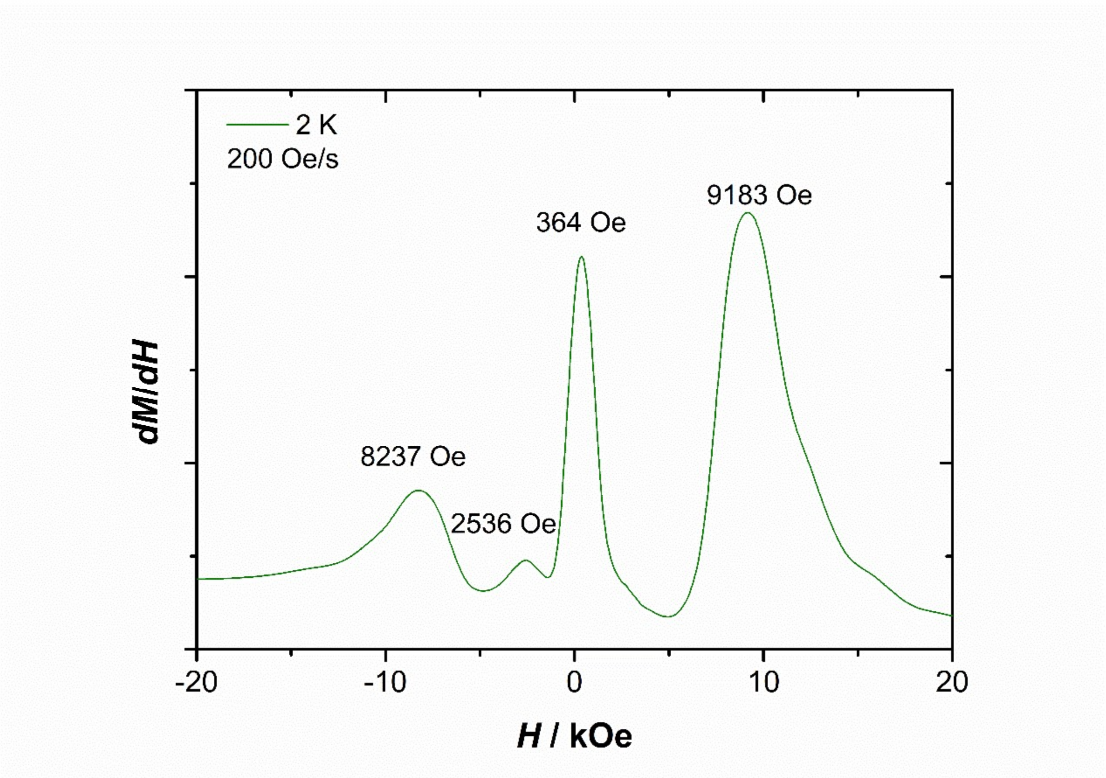

Figure S27. First derivative of the magnetic hysteresis plot for [(η^5-$C_5{}^iPr_5$)Dy(η^5-Cp*)]·0.5hexane at 2 K and a field sweep rate of 200 Oe s^{-1}.

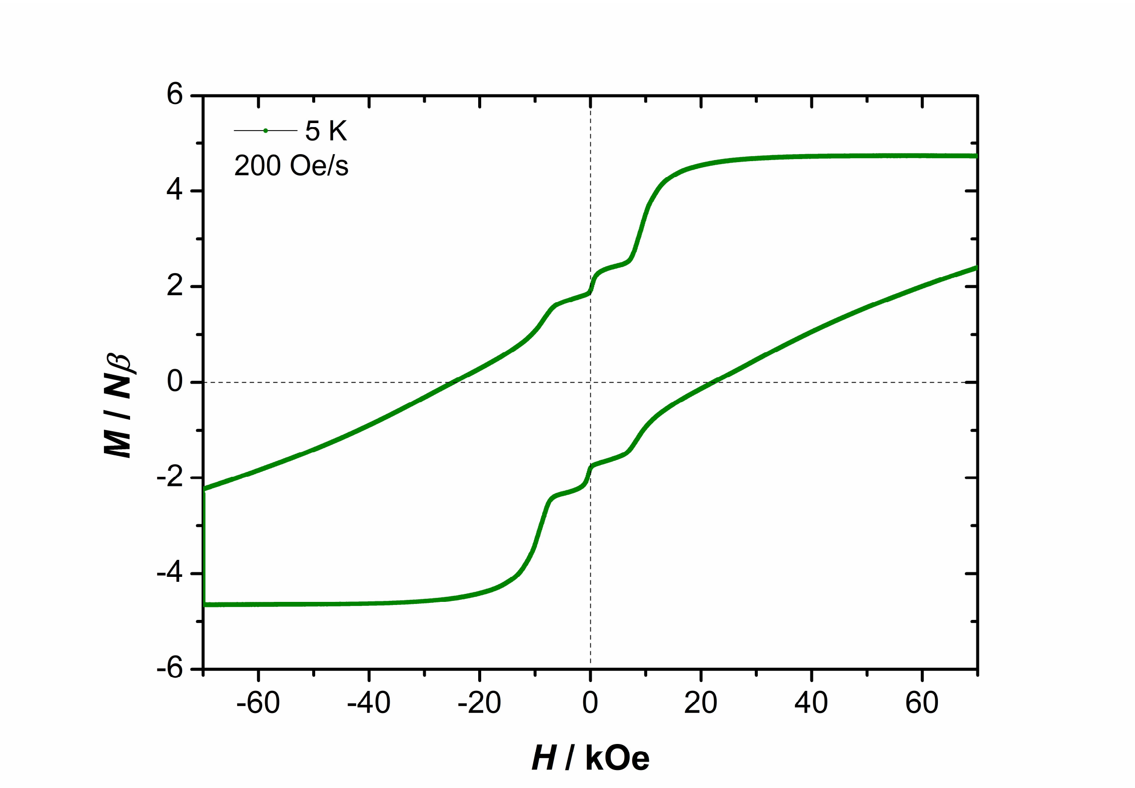

Figure S28. Magnetic hysteresis loops for [(η^5-$C_5{}^iPr_5$)Dy(η^5-Cp*)]·0.5hexane. The data were collected continuously at 5 K using a field sweep speed of 200 Oe s^{-1}.

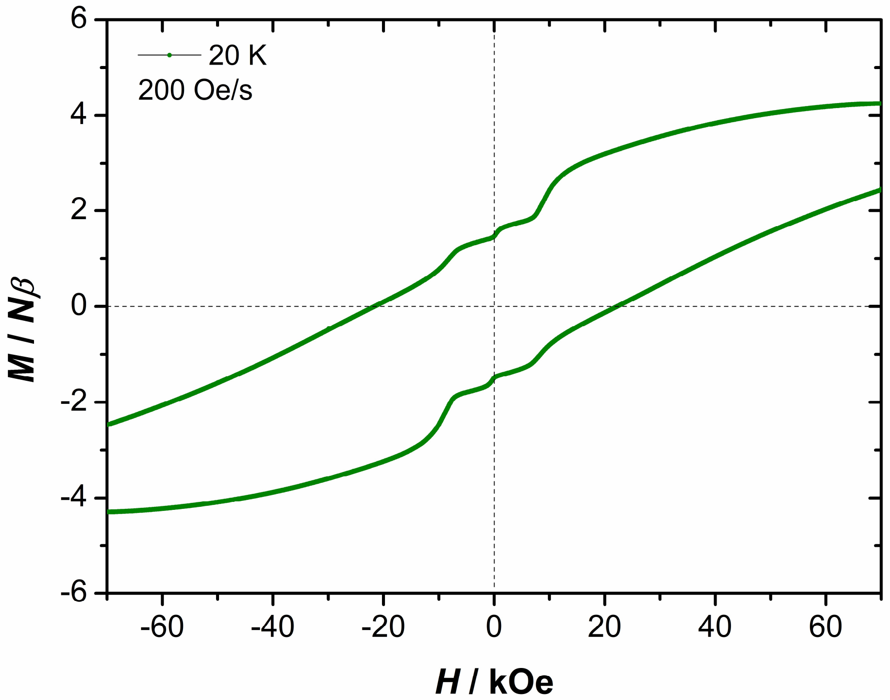

Figure S29. Magnetic hysteresis loops for [(η^5-$C_5{}^iPr_5$)Dy(η^5-Cp*)]·0.5hexane. The data were collected continuously at 20 K using a field sweep speed of 200 Oe s^{-1}.

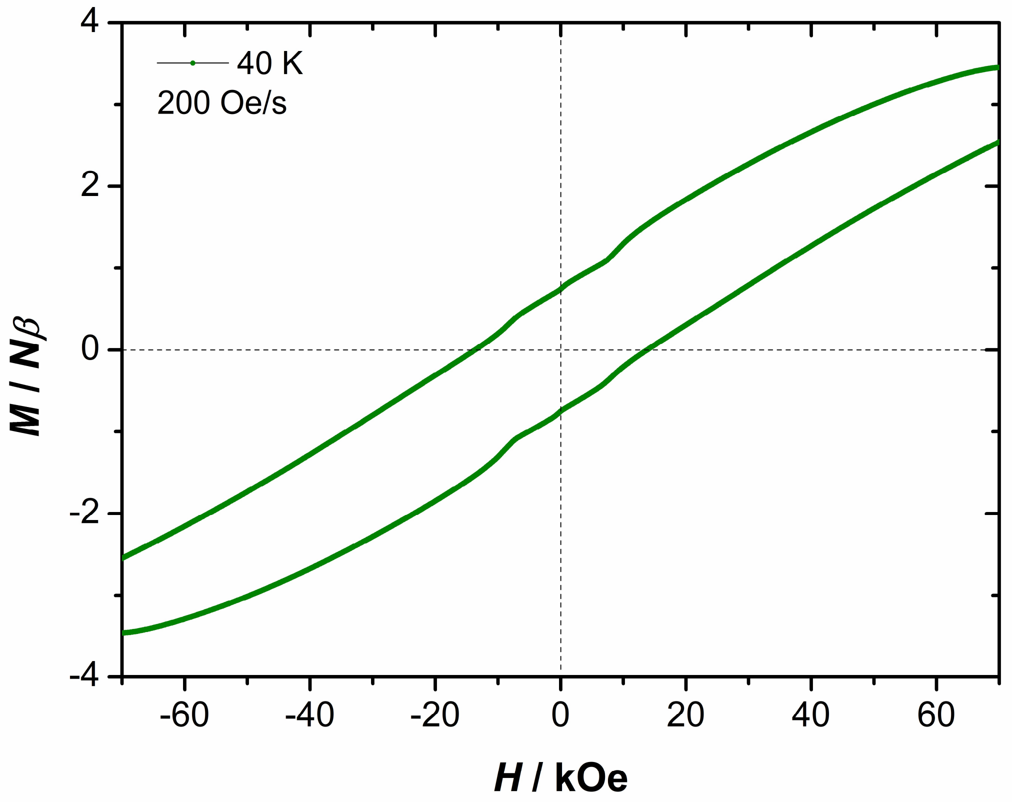

Figure S30. Magnetic hysteresis loops for [(η^5-$C_5{}^iPr_5$)Dy(η^5-Cp*)]·0.5hexane. The data were collected continuously at 40 K using a field sweep speed of 200 Oe s^{-1}.

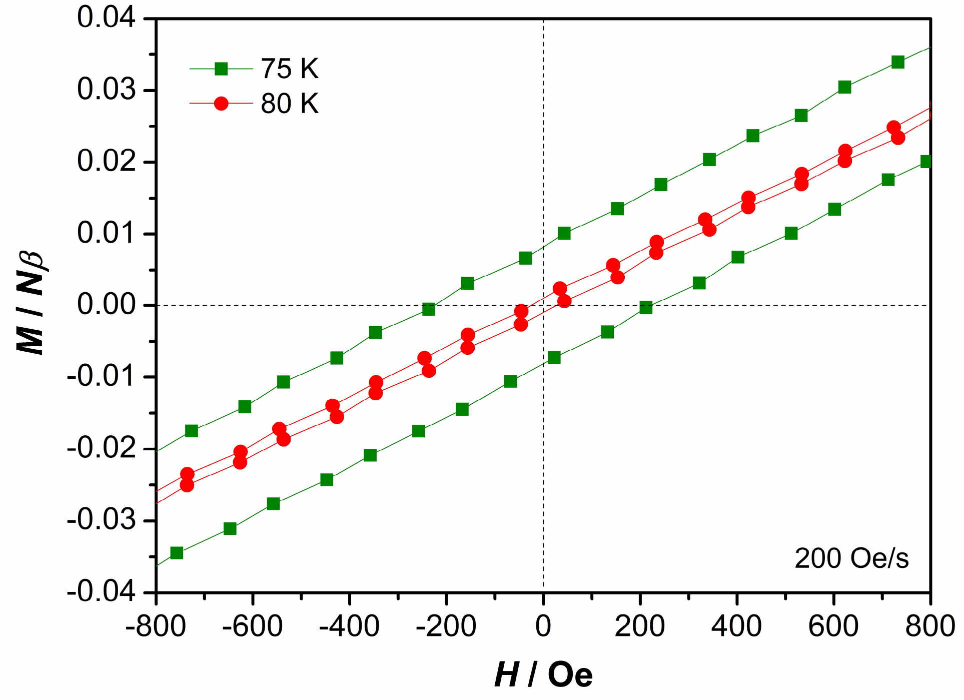

Figure S31. Magnetic hysteresis loops for [(η^5-$C_5{}^iPr_5$)Dy(η^5-Cp*)]·0.5hexane. The data were collected continuously at 75 and 80 K using a field sweep speed of 200 Oe s^{-1}.

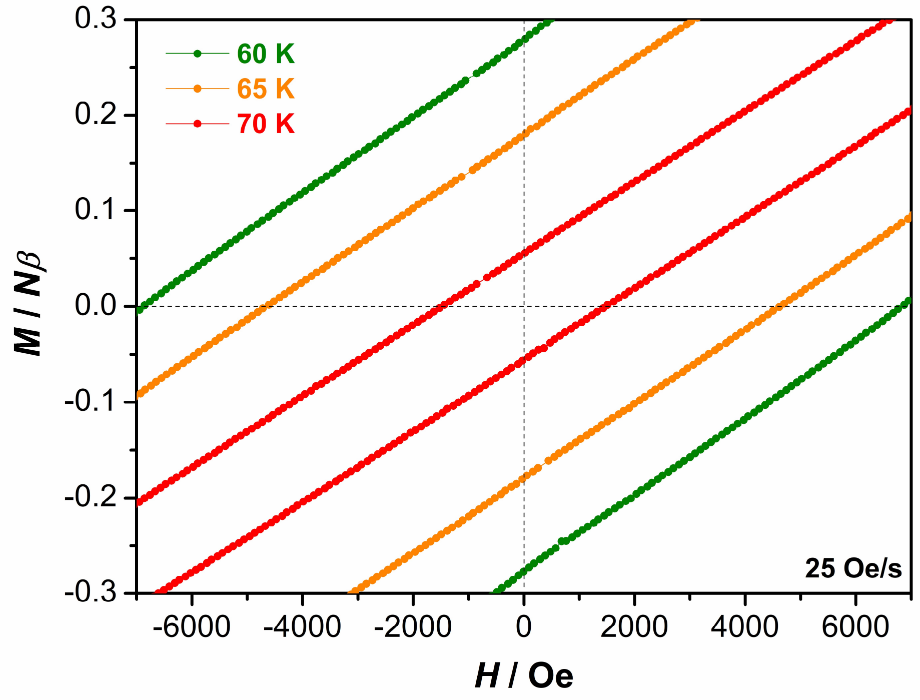

Figure S32. Magnetic hysteresis loops for [(η^5-$C_5{}^iPr_5$)Dy(η^5-Cp*)]·0.5hexane. The data were collected continuously at 60, 65 and 70 K using a field sweep speed of 25 Oe s^{-1}.

Table S5. Remanent magnetization and coercive fields at different temperatures and field sweep rates.

Temperature/K	Field sweep speed / Oe s^{-1}	Remanent magnetization / N_β	Coercive field / Oe
2	200	2.300	31085
5	200	1.925	24823
10	200	1.716	22828
20	200	1.623	21622
30	200	10.75	17582
40	200	0.741	13656
50	200	0.495	10343
60	25	0.279	6721
65	25	0.181	4588
70	25	0.056	1528
75	200	0.008	225
80	200	0.001	25

Computational details

The geometry of [(η^5-$C_5{}^{i}Pr_5$)Dy(η^5-Cp*)] was extracted from the crystal structure. The positions of hydrogen atoms where optimized using density functional theory (DFT) while the positions of heavier atoms were kept frozen to their crystal-structure coordinates. All DFT calculations were carried out using the *Gaussian* 16 program revision C.02.[7] The MWB 4f-in-core effective core potential (ECP) was used for the dysprosium ion with a corresponding valence basis set.[8] 55 electrons were included into the ECP. The remaining atoms were treated with def2-SVP basis sets.[9] The hybrid PBE0 exchange-correlation (XC) functional[10] was used in the geometry optimization, and the CAM-B3LYP range-separated hybrid XC functional[11] was used in time-dependent DFT (TD-DFT) calculations. A total of 100 roots were solved in the TD-DFT calculations.

Multireference calculations were carried out using the *Orca* program version 5.0.4[12] using the state-averaged complete active space self-consistent field (SA-CASSCF) approach.[13] The active space consisted of 14 electrons in 14 orbitals. Scalar relativistic effects were treated with the standard second-order Douglas-Kroll-Hess (DKH) transformation.[14] Electron correlation effects outside the active space were estimated up to second order using the *N*-electron valence state perturbation theory (NEVPT2) in its strongly contracted formulation.[15] In order to reduce computational costs, a single set of orbitals obtained from the state-averaged Fock operator where used for all states in the NEVPT2 calculations. Spin-orbit coupling (SOC) was introduced using the well-established quasi-degenerate perturbation theory (QDPT) approach[16] where the SOC operator is constructed in a basis of the SA-CASSCF eigenstates and diagonalized to yield the final spin-orbit coupled states. The spin-orbit mean-field (SOMF) approach[17] was used in the construction of the SOC operator. SARC2-DKH-QZVP basis[18] was used for the Dy ion, DKH-def2-TZVP basis set was used for C atoms, and DKH-def2-SVP for H atoms.[19] The auxiliary basis set used in the integral transformation was generated using the "AutoAux" feature of Orca.[20] The **g**-tensors and transition magnetic moments were calculated using the SINGLE_ANISO module.[21]

Table S6. TD-DFT transition wavelengths and oscillator strengths calculated for [(η^5-$C_5{}^iPr_5$)Dy(η^5-Cp*)].

λ / nm	f_{osc}	λ / nm	f_{osc}
4218.98	0.0000	203.25	0.0001
3910.90	0.0000	202.70	0.0001
391.94	0.0719	201.11	0.0004
389.78	0.0693	200.91	0.0000
359.40	0.0053	200.79	0.0009
340.52	0.0001	198.49	0.0002
339.56	0.0002	198.35	0.0011
330.10	0.0083	198.13	0.0003
329.34	0.0021	197.83	0.0018
328.53	0.0041	196.89	0.0001
326.83	0.0022	195.05	0.0004
311.45	0.0003	194.90	0.0003
308.26	0.0150	194.79	0.0003
306.66	0.0028	194.74	0.0000
306.43	0.0136	193.13	0.0004
299.93	0.0020	192.83	0.0000
299.70	0.0002	192.56	0.0000
294.03	0.0007	192.39	0.0014
293.52	0.0006	190.15	0.0001
285.23	0.0977	189.59	0.0006
284.97	0.0995	188.82	0.0012
283.49	0.0005	188.72	0.0027
283.02	0.0008	187.44	0.0035
282.47	0.0538	187.09	0.0034
264.51	0.0000	186.85	0.0012
263.40	0.0000	186.50	0.0010
259.98	0.0001	186.28	0.0001
259.90	0.0002	185.53	0.0002
252.68	0.0003	183.48	0.0029
248.86	0.0000	183.10	0.0000
246.68	0.0056	183.01	0.0000
246.33	0.0019	182.45	0.0032
246.15	0.0008	179.50	0.0019
245.54	0.0046	179.15	0.0007
242.50	0.0033	178.85	0.0029
240.15	0.0003	178.35	0.0055
239.78	0.0003	178.04	0.0130

224.23	0.0150	177.87	0.0169
222.80	0.0141	177.68	0.0052
220.69	0.0023	177.12	0.0007
220.04	0.0102	177.09	0.0029
219.38	0.0069	176.92	0.0079
218.26	0.0180	175.45	0.0002
212.30	0.0000	174.75	0.0002
212.02	0.0000	174.43	0.0015
207.67	0.0008	172.88	0.0000
207.12	0.0004	172.19	0.0010
205.63	0.0002	171.88	0.0010
204.74	0.0001	171.24	0.0008
204.40	0.0000	170.54	0.0029

Table S7. Properties of the five lowest quasi-doublets of [(η^5-$C_5{}^iPr_5$)Dy(η^5-Cp*)].

***E* / cm⁻¹**		***g*$_z$** [a]	***θ* /°** [b]
0	0	21.97914	0.0
888	888	18.29514	0.0
1733	1733	14.91948	0.3
2288	2295	17.85564	93.1
2344	2358	5.05254	174.1

[a] In a non-Kramers system the principal *x* and *y* components of the **g** tensor are zero by Griffith's theorem.[22]
[b] The angle between the principal magnetic axis of the given doublet and that of the ground doublet.

Table S8. Magnitudes of transition magnetic moment matrix elements between various states calculated for [(η^5-$C_5{}^iPr_5$)Dy(η^5-Cp*)] in units of Bohr magneton

**Initial quasi-doublet**	**Final quasi-doublet**	**Climbing transition**	**Crossing transition**
1	1	0.000000	0.000000
1	2	1.891333	0.000001
1	3	0.005610	0.000006
1	4	0.002419	0.001337
1	5	0.002018	0.001438
2	2	0.000000	0.000000
2	3	2.530039	0.000038
2	4	0.035707	0.011900
2	5	0.016662	0.018570
3	3	0.000000	0.000000
3	4	0.913175	0.123695
3	5	2.058592	0.713755
3	10	0.000000	0.000000
4	4	0.000000	0.000000
4	5	2.190387	1.208794
5	5	0.000000	0.000000

Table S9. NEVPT2 energies and multiplicities calculated for [(η^5-$C_5{}^iPr_5$)Dy(η^5-Cp*)] before the inclusion of SOC

State	2*S* + 1	*E* / cm^{-1}	State	2*S* + 1	*E* / cm^{-1}
1	7	0.0	55	7	7934.6
2	7	0.1	56	7	8033.6
3	7	2221.5	57	5	8229.3
4	7	2286.0	58	5	8250.3
5	7	2287.5	59	5	8252.4
6	7	2423.9	60	7	8308.9
7	7	2520.6	61	5	8464.6
8	7	2604.9	62	5	8475.5
9	7	2613.7	63	5	8500.7
10	7	2736.6	64	5	8668.6
11	7	2737.4	65	5	8915.6
12	5	3528.6	66	5	8929.2
13	5	3528.9	67	5	8932.5
14	7	3751.8	68	5	9028.2
15	7	3755.6	69	5	9053.6
16	7	3760.2	70	7	9318.7
17	7	3845.3	71	7	9327.3
18	7	3859.9	72	5	9344.8
19	7	3962.8	73	7	9350.7
20	7	4001.4	74	5	9371.8
21	7	4003.5	75	7	9439.6
22	5	4197.2	76	7	9510.3
23	5	4197.2	77	7	9539.9
24	5	4374.0	78	7	9558.6
25	5	4374.7	79	5	9861.6
26	7	4453.8	80	5	9877.5
27	7	4515.7	81	7	9890.7
28	7	4536.1	82	7	9915.3
29	7	4566.6	83	7	9989.2
30	7	4572.3	84	7	10082.9
31	5	4640.8	85	5	10144.1
32	5	4645.6	86	5	10149.6
33	7	4803.8	87	5	10152.5
34	7	4839.9	88	7	10167.6
35	7	4865.1	89	7	10192.0
36	7	4942.9	90	7	10333.1
37	5	5087.9	91	7	10342.7

38	5	5102.1	92	7	10510.4
39	7	5176.7	93	5	10687.2
40	7	5196.4	94	5	10687.6
41	7	5206.8	95	5	13347.9
42	5	5261.0	96	5	13349.3
43	7	5334.9	97	5	13365.7
44	7	5379.2	98	5	13366.6
45	5	7103.9	99	5	13448.0
46	5	7112.1	100	5	13448.6
47	5	7498.6	101	5	13990.5
48	5	7502.0	102	5	14043.3
49	5	7731.4	103	5	14047.9
50	5	7741.2	104	5	14182.7
51	5	7830.1	105	5	14338.7
52	5	7830.5	106	5	14434.6
53	7	7878.5	107	5	14441.5
54	7	7887.8	108	5	14571.9

Table S10. QDPT eigenvalues calculated for [(η^5-$C_5{}^iPr_5$)Dy(η^5-Cp*)] in cm^{-1}.

0.00	5942.56	8813.43	10762.42	12349.39	13794.24	15504.15	17585.91
0.00	5943.76	8818.49	10806.24	12355.32	13843.28	15525.76	17692.08
888.33	6034.00	8879.23	10852.38	12360.15	13915.37	15545.06	17708.12
888.33	6053.75	8879.73	10862.58	12368.02	13923.33	15560.04	17801.33
1732.87	6081.17	8896.15	10914.72	12388.19	13962.02	15738.31	17812.69
1732.92	6084.38	8902.76	10915.98	12456.51	13967.34	15753.30	17818.20
2288.00	6093.71	8904.36	10933.53	12458.31	13983.45	15840.74	17890.35
2295.24	6102.72	8910.31	10948.25	12462.08	14009.70	15952.94	17982.35
2343.87	6105.89	8924.13	10952.30	12494.63	14077.06	15954.14	18031.98
2358.02	6132.73	8935.20	10955.16	12501.58	14116.74	15967.99	18044.78
2360.50	6160.06	9021.93	10972.33	12528.15	14189.90	15969.11	18046.46
2391.26	6201.83	9028.02	10978.27	12534.87	14197.48	16034.37	18140.52
2399.44	6208.81	9033.04	10998.79	12543.97	14212.88	16043.73	18164.64
2488.18	6249.58	9039.24	11166.56	12544.84	14229.35	16048.48	18175.76
2489.68	6277.92	9073.65	11169.72	12577.68	14232.37	16056.05	18209.76
2544.20	6295.52	9090.64	11215.85	12602.65	14243.58	16074.08	18233.13
2546.80	6318.22	9140.44	11226.26	12611.83	14266.76	16151.52	18351.17
3187.74	6325.86	9143.53	11288.83	12613.10	14269.92	16162.48	18355.51
3187.76	6335.42	9192.01	11310.49	12627.75	14357.91	16186.25	18358.28
3282.09	6344.19	9207.91	11322.42	12647.71	14374.69	16215.48	18362.10
3303.79	6363.51	9228.89	11332.28	12679.65	14481.50	16222.99	18367.83
3314.49	6382.74	9331.82	11348.70	12693.09	14496.58	16243.44	18373.11
3317.79	6385.85	9344.72	11367.72	12698.02	14504.25	16248.27	18386.86
3333.29	6417.88	9373.49	11399.87	12754.03	14591.87	16281.37	18411.49
3338.77	6425.09	9395.47	11401.38	12762.45	14615.77	16289.75	18432.38
3343.10	6496.82	9401.85	11430.31	12766.68	14617.66	16308.23	18457.73
3356.73	6500.13	9454.46	11441.70	12770.32	14646.70	16312.91	18460.56
3360.84	6581.79	9487.20	11459.27	12786.15	14653.36	16335.63	18476.74
3447.70	6636.74	9495.48	11487.68	12795.02	14661.49	16347.49	18508.63
3448.60	6652.36	9499.68	11543.06	12824.95	14664.80	16352.41	18553.10
3484.01	6675.71	9602.57	11562.95	12855.63	14721.87	16404.15	18568.61
3484.56	6711.56	9632.92	11581.06	12889.24	14739.67	16418.42	18582.93
3745.70	6714.48	9663.10	11620.94	12906.41	14775.21	16423.91	18651.02
3748.48	6727.67	9678.93	11633.87	12910.30	14780.73	16442.49	18774.33
3770.98	6736.74	9688.06	11662.00	12987.37	14784.91	16467.14	18774.72
3776.16	6787.56	9715.91	11668.75	13014.22	14789.63	16549.85	18792.91
3789.29	6792.90	9716.64	11685.51	13016.57	14795.31	16561.45	18821.67
3796.05	6800.69	9748.61	11697.10	13027.05	14801.17	16604.35	18826.06

3887.04	7138.59	9751.95	11703.66	13068.58	14812.40	16623.30	18842.43
3890.38	7154.25	9766.13	11710.59	13069.61	14826.34	16637.47	18855.47
3894.35	7331.65	9772.34	11723.01	13105.94	14830.62	16643.85	18935.63
3904.17	7345.75	9780.55	11726.60	13111.27	14836.43	16653.18	18951.70
3911.49	7379.56	9786.12	11773.72	13133.67	14861.25	16656.54	19018.34
3935.31	7655.93	9791.95	11781.67	13142.69	14864.63	16662.83	19066.43
3940.61	7656.93	9797.82	11790.54	13152.21	14895.29	16676.86	19081.30
4180.27	7663.67	9874.69	11804.66	13161.37	14901.51	16686.05	19104.44
4184.37	7735.02	9875.37	11811.55	13168.08	14908.95	16691.73	19139.20
4230.88	7774.46	9904.71	11820.54	13178.54	14925.83	16702.29	19173.80
4231.81	7802.83	9904.85	11828.26	13186.79	14944.27	16705.24	19331.18
4314.25	7807.90	10008.38	11846.06	13191.03	14955.57	16779.81	19336.44
4317.41	7899.25	10010.04	11851.75	13260.43	14967.70	16795.06	19364.39
4486.07	7903.67	10046.95	11857.36	13275.15	14975.94	16827.28	20079.67
4538.26	7904.60	10048.94	11879.37	13285.99	15009.21	16832.16	20082.39
4579.08	7909.47	10066.49	11882.20	13329.30	15020.65	16849.97	20167.87
4591.11	8166.22	10071.67	11890.16	13359.39	15061.04	16947.51	20172.83
4713.06	8170.32	10078.95	11903.15	13376.04	15097.53	16954.51	20203.59
4716.66	8286.78	10130.55	11910.71	13377.79	15107.50	17003.11	20204.73
4830.12	8322.66	10137.30	11914.63	13394.25	15115.85	17014.15	20338.99
4835.45	8343.51	10177.92	11922.27	13398.66	15132.17	17059.23	20443.51
4879.37	8356.32	10194.52	11927.12	13413.64	15153.57	17064.85	20448.26
5013.86	8372.12	10200.38	11944.06	13426.01	15158.18	17079.34	20466.13
5019.85	8376.70	10217.74	12033.92	13447.09	15172.50	17116.98	20585.01
5072.06	8389.07	10317.18	12048.34	13452.91	15181.05	17161.81	20643.58
5072.68	8398.81	10343.12	12055.24	13481.18	15202.75	17168.29	20691.05
5125.64	8407.48	10385.92	12062.41	13496.71	15212.22	17187.56	20694.58
5130.93	8442.04	10411.18	12093.05	13508.39	15230.67	17199.87	21354.91
5218.97	8454.01	10427.58	12100.67	13530.85	15242.45	17216.75	21368.50
5222.99	8460.37	10444.58	12102.20	13537.04	15250.22	17248.44	21537.11
5288.10	8475.50	10449.62	12110.33	13548.70	15252.07	17279.67	21540.59
5293.52	8478.05	10455.83	12118.10	13565.34	15254.04	17292.58	21546.93
5388.82	8493.22	10492.51	12173.90	13585.22	15259.48	17308.56	21644.16
5404.31	8500.68	10508.42	12215.85	13600.37	15263.37	17315.24	21761.20
5425.95	8521.46	10512.86	12218.46	13604.85	15264.21	17337.04	21769.11
5475.94	8527.12	10540.47	12226.07	13630.49	15279.76	17377.80	21853.24
5489.94	8533.87	10586.90	12242.36	13683.93	15308.36	17400.51	22066.98
5499.83	8622.31	10622.49	12248.07	13732.23	15316.20	17408.80	22230.82
5510.55	8623.57	10630.44	12253.45	13750.47	15331.09	17421.44	22235.89

5544.28	8646.03	10665.62	12272.96	13757.00	15397.77	17441.11	22536.57
5551.61	8651.56	10672.79	12298.23	13761.96	15462.00	17464.62	22665.49
5581.97	8760.68	10689.62	12312.52	13766.59	15466.66	17479.06	22729.97
5589.09	8783.09	10692.77	12337.01	13768.90	15496.65	17530.73	22745.39

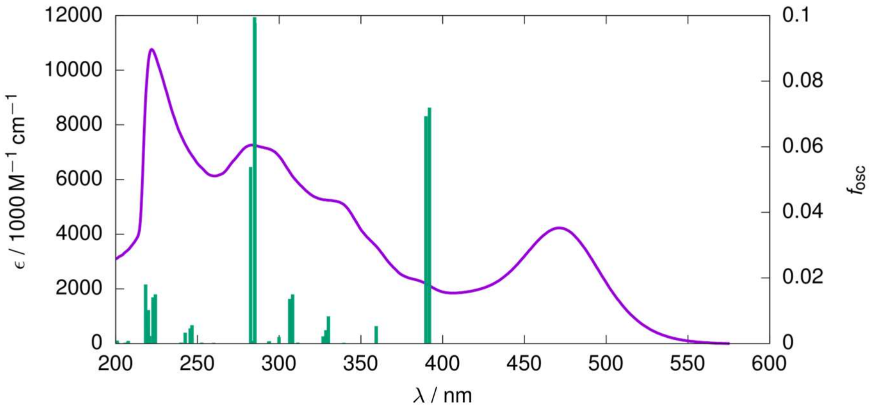

Figure S33. Experimental (violet) and calculated (green) UV-vis spectra of [(η^5-$C_5{}^iPr_5$)Dy(η^5-Cp*)]·0.5hexane.

References

Guo, F.-S.; Day, B. M.; Chen, Y.-C.; Tong, M.-L.; Mansikkamäki, A.; Layfield, R. A. Magnetic Hysteresis up to 80 Kelvin in a Dysprosium Metallocene Single-Molecule Magnet. *Science* **2018**, *362*, 1400-1403.

Evans, W. J.; Kozimor, S. A.; Ziller, J. W.; Kaltsoyannis, N. Structure, Reactivity, and Density Functional Theory Analysis of the Six-Electron Reductant, $[(C_5Me_5)_2U]_2(\mu\text{-}\eta^6:\eta^6\text{-}C_6H_6)$, Synthesized via a New Mode of $(C_5Me_5)_3M$ Reactivity. *J. Am. Chem. Soc.* **2004**, *126*, 14533-14547.

Dolomanov, O. V; Bourhis, L. J.; Gildea, R. J.; Howard, J. A. K.; Puschmann, H. OLEX2: A Complete Structure Solution, Refinement and Analysis Program. *J. Appl. Crystallogr.* **2009**, *42*, 339-341.

Sheldrick, G. M. A Short History of SHELX. *Acta Cryst. A* **2008**, *64*, 112-122.

Sheldrick, G. M. Crystal Structure Refinement with SHELXL. *Acta Cryst. C* **2015**, *71*, 3-8.

Bain, G. A.; Berry, J. F. Diamagnetic Corrections and Pascal's Constants. *J. Chem. Educ.* **2008**, *85*, 532.

Gaussian 16, Revision C.02, M. J. Frisch, G. W. Trucks, H. B. Schlegel, G. E. Scuseria, M. A. Robb, J. R. Cheeseman, G. Scalmani, V. Barone, G. A. Petersson, H. Nakatsuji, X. Li, M. Caricato, A. V. Marenich, J. Bloino, B. G. Janesko, R. Gomperts, B. Mennucci, H. P. Hratchian, J. V. Ortiz, A. F. Izmaylov, J. L. Sonnenberg, D. Williams-Young, F. Ding, F. Lipparini, F. Egidi, J. Goings, B. Peng, A. Petrone, T. Henderson, D. Ranasinghe, V. G. Zakrzewski, J. Gao, N. Rega, G. Zheng, W. Liang, M. Hada, M. Ehara, K. Toyota, R. Fukuda, J. Hasegawa, M. Ishida, T. Nakajima, Y. Honda, O. Kitao, H. Nakai, T. Vreven, K. Throssell, J. A. Montgomery, Jr., J. E. Peralta, F. Ogliaro, M. J. Bearpark, J. J. Heyd, E. N. Brothers, K. N. Kudin, V. N. Staroverov, T. A. Keith, R. Kobayashi, J. Normand, K. Raghavachari, A. P. Rendell, J. C. Burant, S. S. Iyengar, J. Tomasi, M. Cossi, J. M. Millam, M. Klene, C. Adamo, R. Cammi, J. W. Ochterski, R. L. Martin, K. Morokuma, O. Farkas, J. B. Foresman, and D. J. Fox, *Gaussian, Inc.*, Wallingford CT, **2016**.

(a) M. Dolg, H. Stoll, A. Savin, H. Preuß. *Theor. Chim. Acta.* **1989**, *75*, 173–194; (b) D. Andrae, U. Häußermann, M. Dolg, H. Stoll, H. Preuß. *Theor. Chim. Acta.* **1990**, *77*, 123–141.

(a) F. Weigend, R. Ahlrichs. *Phys. Chem. Chem. Phys.* **2005**, *7*, 3297–3305; (b) A. Schäfer, H. Horn, R. Ahlrichs. *J. Chem. Phys.* **1992**, *97*, 2571–2577.

(a) J. P. Perdew, K. Burke, M. Ernzerhof. *Phys. Rev. Lett.*, **1996**, *77*, 3865–3868; (b) J. P. Perdew, K. Burke, M. Ernzerhof. *Phys. Rev. Lett.*, **1996**, *78*, 1396; (c) M. Ernzerhof, G. E. Scuseria. *J. Chem. Phys.* **1999**, 119, 5029–5036; (d) C. Adamo, V. Barone. *J. Chem. Phys.* **1999**, *110*, 6158–6170.

(a) T. Yanai, D. P. Tew, N. C. Handy. *Chem. Phys. Lett.* **2004**, *393*, 51–57; (b) A. D. Becke. *Phys. Rev. A.* **1988**, *38*, 3098–3100; c) C. Lee, W. Yang, R. G. Parr. *Phys. Rev. B.* **1988**, *37*, 785–789.

(a) F. Neese. *WIREs Comput. Mol. Sci.* **2022**, *12*, e1606; (b) F. Neese. *J. Comp. Chem.* **2022**, *44*, 381–396; c) F. Neese, F. Wennmohs, U. Becker, C. Riplinger. *J. Chem. Phys.* **2020**, *152*, 224108; d) F. Neese. *WIREs Comput. Mol. Sci.* **2017**, *8*, e1327.

(a) B. O. Roos in *Advances in Chemical Physics*, *Ab Initio Methods in Quantum Chemistry II*, Vol. 69 (Ed.: K. P. Lawley), Wiley, New York, **1987**, pp. 399–455; (b) P. Siegbahn, A. Heiberg, B. Roos, B. Levy. *Phys. Scripta*, **1980**, *21*, 323–327; (c) B. O. Roos, P. R. Taylor, P. E. M. Siegbahn. *Chem. Phys.*, **1980**, *48*, 157–173; (d) P. E. M. Siegbahn, J. Almlöf, A. Heiberg, B. Roos. *J. Chem. Phys.*, **1981**, *74*, 2384–2396; (e) B. O. Roos, R. Lindh, P. Å. Malmqvist, V. Veryazov, P.-O. Widmark. *Multiconfigurational Quantum Chemistry.* Wiley, Hoboken, NJ, **2016**.

(a) M. Douglas, N. M. Kroll. *Ann. Phys.* **1974**, *82*, 89–155; b) B. A. Heß. *Phys. Rev. A.* **1986**, *33*, 3742–3748.

(a) C. Angeli, R. Cimiraglia, S. Evangelisti, T. Leininger, J.-P. Malrieu. *J. Chem. Phys.* **2001**, *114*, 10252–10264; (b) C. Angeli, R. Cimiraglia, J.-P. Malrieu. *Chem. Phys. Lett.* **2001**, *350*, 297–305; (c) C. Angeli, R. Cimiraglia, J.-P. Malrieu. *J. Chem. Phys.* **2002**, *117*, 9138–9153.

(a) F. Neese, T. Petrenko, D. Ganyushin, G. Olbrich. *Coord. Chem. Rev.* **2007**, *251*, 288–327; (b) M. Atanasov, D. Aravena, E. Suturina, E. Bill, D. Maganas, F. Neese. *Coord. Chem. Rev.* **2015**, *289–290*, 177–214.

(a) F. Neese. *J. Chem. Phys.* **2005**, *122*, 034107; (b) A. Berning, M. Shcweizer, H.-J. Werne, P. J. Knowles, P. Palmieri. *Mol. Phys.* **2000**, *98*, 1823–1833; (c) B. A. Heß, C. M. Marian, U. W. Wahlgren, O. Gropen. *Chem. Phys. Lett.* **1996**, *251*, 365–371.

D. Aravena, F. Neese, D. A. Pantasiz. *J. Chem. Theory Comput.* **2016**, *12*, 1148–1156.

(a) D. A. Pantazis, X.-Y. Chen, C. R. Landis, F. Neese. *J. Chem. Theory Comput.* **2008**, *4*, 908–919. G. L. Stoychev, A. A. Auer, F. Neese. *J. Chem. Theory Comput.* **2017**, *13*, 554–562.

(a) L. F. Chibotaru, L. Ungur. *J. Chem. Phys.*, **2012**, *137*, 064112; (b) L. Ungur, L. F. Chibotaru. Computational *Modelling of Magnetic Properties of Lanthanide Compounds* in *Lanthanide and Actinides in Molecular Magnetism.* Eds. R. A. Layfield, M. Murugesu. Wiley,-VHC, Weinheim, Germany, **2015**; (c) L. Ungur, M. Thewissen, J.-P. Costes, W. Wernsdorfer, L. F. Chibotaru. *Inorg. Chem.*, **2013**, *52*, 6328–6337.
J. S. Griffith. *Phys. Rev.* **1963**, *132*, 316–319.
